# Akap5 links synaptic dysfunction to neuroinflammatory signaling in a mouse model of infantile neuronal ceroid lipofuscinosis

**DOI:** 10.3389/fnsyn.2024.1384625

**Published:** 2024-05-10

**Authors:** Kevin P. Koster, Zach Fyke, Thu T. A. Nguyen, Amanda Niqula, Lorena Y. Noriega-González, Kevin M. Woolfrey, Mark L. Dell’Acqua, Stephanie M. Cologna, Akira Yoshii

**Affiliations:** ^1^Department of Anatomy and Cell Biology, University of Illinois at Chicago, Chicago, IL, United States; ^2^Department of Chemistry, University of Illinois at Chicago, Chicago, IL, United States; ^3^Department of Pharmacology, University of Colorado School of Medicine, Aurora, CO, United States; ^4^Department of Pediatrics, University of Illinois at Chicago, Chicago, IL, United States; ^5^Department of Neurology, University of Illinois at Chicago, Chicago, IL, United States

**Keywords:** palmitoylation, depalmitoylation, PPT1, synaptic scaling, neuroinflammation, lipofuscinosis, Akap5

## Abstract

Palmitoylation and depalmitoylation represent dichotomic processes by which a labile posttranslational lipid modification regulates protein trafficking and degradation. The depalmitoylating enzyme, palmitoyl-protein thioesterase 1 (PPT1), is associated with the devastating pediatric neurodegenerative condition, infantile neuronal ceroid lipofuscinosis (CLN1). CLN1 is characterized by the accumulation of autofluorescent lysosomal storage material (AFSM) in neurons and robust neuroinflammation. Converging lines of evidence suggest that in addition to cellular waste accumulation, the symptomology of CLN1 corresponds with disruption of synaptic processes. Indeed, loss of Ppt1 function in cortical neurons dysregulates the synaptic incorporation of the GluA1 AMPA receptor (AMPAR) subunit during a type of synaptic plasticity called synaptic scaling. However, the mechanisms causing this aberration are unknown. Here, we used the *Ppt1^−/−^* mouse model (both sexes) to further investigate how Ppt1 regulates synaptic plasticity and how its disruption affects downstream signaling pathways. To this end, we performed a palmitoyl-proteomic screen, which provoked the discovery that Akap5 is excessively palmitoylated at *Ppt1^−/−^* synapses. Extending our previous data, *in vivo* induction of synaptic scaling, which is regulated by Akap5, caused an excessive upregulation of GluA1 in *Ppt1^−/−^* mice. This synaptic change was associated with exacerbated disease pathology. Furthermore, the Akap5- and inflammation-associated transcriptional regulator, nuclear factor of activated T cells (NFAT), was sensitized in *Ppt1^−/−^* cortical neurons. Suppressing the upstream regulator of NFAT activation, calcineurin, with the FDA-approved therapeutic FK506 (Tacrolimus) modestly improved neuroinflammation in *Ppt1^−/−^* mice. These findings indicate that the absence of depalmitoylation stifles synaptic protein trafficking and contributes to neuroinflammation via an Akap5-associated mechanism.

## 1 Introduction

Proteostasis is a fundamental molecular mechanism whereby quality, quantity, and distribution of proteins are rigorously controlled through synthesis, trafficking, and degradation (Labbadia and Morimoto, 2014; Winckler et al., 2018; Liang, 2019). Lysosomal digestion is one of several mechanisms responsible for protein degradation (Nandi et al., 2006; Walter and Ron, 2011; Cao and Kaufman, 2012; Nixon, 2013; Xu and Ren, 2015; Lie and Nixon, 2018), playing a critical role in the breakdown of lipid-modified proteins, such as palmitoylated proteins.

Protein palmitoylation is a reversible posttranslational lipid modification that impacts most, if not all, aspects of proteostasis (Washbourne, 2004; Fukata and Fukata, 2010; Levental et al., 2010). Intriguingly, palmitoylation is particularly prevalent among synaptic proteins (Sanders et al., 2015), including synaptic scaffolds and neurotransmitter receptor subunits (Fukata and Fukata, 2010). Palmitoylation is conducted by a class of over 20 enzymes called protein acyltransferases (PATs) (Fukata et al., 2004; Noritake et al., 2009; Woolfrey et al., 2015; Tabaczar et al., 2017). Conversely, depalmitoylation is performed by a restricted group of enzymes that includes palmitoyl-protein thioesterase 1 (PPT1) (Won et al., 2018).

PPT1 is considered an endo-lysosomal enzyme, though it also has putative substrates within the synaptic cytosol (Camp et al., 1994; Koster and Yoshii, 2019; Gorenberg et al., 2022) and can depalmitoylate proteins extracellularly, in the synaptic cleft, for instance (Gorenberg et al., 2022). Therefore, PPT1 can influence the trafficking and degradation of many proteins across multiple cellular compartments. Accordingly, mutations in the gene encoding PPT1, *CLN1*, cause infantile neuronal ceroid lipofuscinosis (CLN1), a fatal pediatric neurodegenerative disease that presents with sensory loss, motor regression, and seizure (Vesa et al., 1995; Haltia, 2003; Nita et al., 2016). While histopathological hallmarks of CLN1 include the robust accumulation of autofluorescent lysosomal storage material (AFSM) and gliosis (Nita et al., 2016), accumulating evidence suggests a role for PPT1 in the regulation of synaptic protein function (Finn et al., 2012; Koster et al., 2019, 2023; Sapir et al., 2019; Gorenberg et al., 2022).

Our recent study (Koster et al., 2023) demonstrated that PPT1-mediated depalmitoylation regulates a form of synaptic plasticity termed synaptic scaling (O’Brien et al., 1998; Turrigiano et al., 1998). Specifically, loss of Ppt1 causes an excessive upregulation of GluA1 during synaptic upscaling in cortical neurons (Koster et al., 2023). However, the mechanisms by which loss of PPT1 affects synaptic AMPAR trafficking remain incompletely understood. Additionally, it is unclear how abnormal neurotransmission translates into more proximal pathogenic features of CLN1, like neuroinflammation and neuronal death.

Therefore, the goal of the current study was to investigate the link between the disrupted function of AMPARs and the downstream consequences contributing to the pathogenesis of CLN1. An unbiased palmitoyl-proteomic screen using the *Ppt1^−/−^* mouse model revealed that Ppt1 can regulate the proteostasis of AMPARs not only by affecting the GluA1 palmitoylation state (Koster et al., 2023), but also the palmitoylation state of the AMPAR-associated molecule, Akap5. Furthermore, we provide a mechanistic link between aberrant synaptic plasticity and neuroinflammation through an Akap5-associated pathway in CLN1. Finally, we demonstrate that pharmacologically targeting this pathway slightly ameliorates neuroinflammation in *Ppt1^−/−^* mice.

## 2 Materials and methods

### 2.1 Animals, group allocation, and data handling

All animal procedures were performed in accordance with the guidelines of the University of Illinois at Chicago Institutional Animal Care and Use Committee. *Ppt1^+/−^* (heterozygous) mice were maintained on 12-h light/dark cycle with food and water *ad libitum* and bred to generate *Ppt1^−/−^* (CLN1), *Ppt1^+/−^*, and *Ppt1^+/+^* (wild-type, WT) animals (Gupta et al., 2001). Animals of both sexes were allocated to groups based on their genotype (i.e., WT or *Ppt1^−/−^*), without specific regard to equally balancing the number of males or females across groups. Therefore, males and females were used in roughly equal proportion. Imaging data were acquired randomly (no criteria were used for selecting cells, view fields, etc. except where anatomically necessary). All data were acquired and maintained without descriptive naming/labeling to ease randomization. Severe seizure, defined as an animal suffering from either prolonged (3–5 min) epileptic episodes or a failure to recover normal locomotor activity from such episodes, was considered as a humane endpoint criterion and prompted euthanasia but was not systematically quantified.

### 2.2 Brain fractionation, biochemical assays from tissue samples, and immunoblotting

WT and *Ppt1^−/−^* brains were collected as previously (Koster et al., 2019, 2023). For biochemical analysis of total protein content, isolated visual cortices from *Ppt1^−/−^* and WT animals were homogenized in ice-cold synaptosome buffer (320 mM sucrose, 1 mM EDTA, 4 mM HEPES, pH 7.4) containing 1× protease inhibitor cocktail (Roche), 1× phosphatase inhibitor cocktail (Roche), and 1 mM PMSF using 30 strokes in a Dounce homogenizer. Aliquots of lysates were stored at −80°C, and the remaining sample was used for synaptosome preparation, which was performed as follows: whole lysates were centrifuged at 1000 × *g* to remove cellular debris, after which supernatant was centrifuged at 12,000 × *g* for 15 min to generate pellet P2. The P2 pellet was resuspended in synaptosome buffer and centrifuged at 18,000 × *g* for 15 min to produce the synaptosomal membrane fraction, LP1, referred to hereafter as synaptosomes.

For immunoblotting, the protein concentration of each sample was determined using BCA protein assay (Pierce). Samples were then brought to 20 μg total protein in 2× Laemmli buffer containing 10% β-mercaptoethanol (Bio-Rad), heated at 70°C for 10 min, and loaded into 4–20% precast gels (Bio-Rad) for electrophoresis (130 V, 1.5–2 h). Proteins were wet-transferred to PVDF membranes (Immobillon-P, Millipore), blocked in TBS, pH 7.4, containing 5% non-fat milk and 0.1% Tween-20 (TBS-T + 5% milk). Membranes were incubated in primary antibody solutions containing 2% BSA in TBS-T for 2 h at room temperature (RT) or overnight at 4°C. Primary antibodies were used according to Table 4. Membranes were then incubated with appropriate secondary, HRP-conjugated antibodies (Jackson ImmunoResearch) at either 1:1,000 or 1:5,000 for 1 h at RT before washing three times with TBS-T. Visualization and quantification were performed using the Pierce SuperSignal ECL substrate and Odyssey-FC chemiluminescent imaging station (LI-COR). Signal density for each synaptic protein was measured using the LI-COR software, Image Studio Lite (version 5.2), and normalized to the signal density for β-actin loading control for each lane. Two technical replicates for each experiment were averaged together to get one n.

**Table 1:** Statistics for Sholl measurements in Figure 4D.

	P33							
	WT vs. *Ppt1^−/−^*		WT vs. DR-WT		WT vs. DR-*Ppt1^−/−^*		*Ppt1^−/−^* vs. *Ppt1^−/−^*	
Distance from soma (mm)	Significant?	*p*-value (FDR-corrected)	Significant?	*p*-value (FDR-corrected)	Significant?	*p*-value (FDR-corrected)	Significant?	*p*-value (FDR-corrected)
5	No	0.218615	No	0.731765	No	0.342738	No	0.764286
7	No	0.115316	No	0.665366	No	0.023971	No	0.507645
9	No	0.023024	No	0.757519	No	0.070949	No	0.632289
11	No	0.016119	No	0.749431	No	0.320358	No	0.16471
13	No	0.443392	No	0.433162	No	0.949456	No	0.435926
15	No	0.169529	No	0.885876	No	0.678793	No	0.330704
17	No	0.019493	No	0.203353	No	0.021276	No	0.923168
19	No	0.238231	No	0.184467	No	0.193691	No	0.972754
21	No	0.103026	No	0.081512	No	0.013395	No	0.415518
23	No	0.088268	No	0.065691	No	0.005041	No	0.238345
25	No	0.017733	No	0.207134	No	0.005962	No	0.522911
27	No	0.100663	No	0.332808	No	0.008963	No	0.1873
29	No	0.042891	No	0.127728	No	0.001193	No	0.161231
31	No	0.093855	No	0.056172	No	0.005948	No	0.184524
33	No	0.095839	No	0.068832	No	0.006357	No	0.199908
35	No	0.756193	No	0.084214	No	0.033589	No	0.072587
37	No	0.460413	No	0.448059	No	0.083245	No	0.233528
39	No	0.996764	No	0.820076	No	0.545421	No	0.508027
41	No	0.151357	No	0.95319	No	0.895777	No	0.214591
43	No	0.177366	No	0.98	No	0.47443	No	0.042373
45	No	0.174571	No	0.374394	No	0.757919	No	0.101548
47	No	0.220771	No	0.307062	No	0.59165	No	0.069981
49	No	0.394463	No	0.218616	No	0.981856	No	0.378607
51	No	0.070006	No	0.428862	No	0.319585	No	0.453046
53	No	0.067068	No	0.969558	No	0.512291	No	0.175332
55	No	0.037601	No	0.34815	No	0.126837	No	0.411259
57	No	0.060524	No	0.210592	No	0.289357	No	0.10001
59	No	0.056984	No	0.210592	No	n/a	No	0.073701
61	No	0.085294	No	0.210592	No	n/a	No	0.105944
63	No	0.152835	No	0.210592	No	n/a	No	0.179282
65	No	0.17833	No	n/a	No	n/a	No	0.206114
67	No	0.289357	No	n/a	No	n/a	No	0.319632
69	No	0.289357	No	n/a	No	n/a	No	0.319632

	P42							
	WT vs. *Ppt1^−/−^*		WT vs. DR-WT		WT vs. DR-*Ppt1^−/−^*		*Ppt1^−/−^* vs. *Ppt1^−/−^*	
Distance from soma (mm)	Significant?	*p*-value (FDR-corrected)	Significant?	*p*-value (FDR-corrected)	Significant?	*p*-value (FDR-corrected)	Significant?	*p*-value (FDR-corrected)
5	No	0.396741	No	0.921723	No	0.258172	No	0.785052
7	No	0.248326	No	0.195573	No	0.429474	No	0.51606
9	No	0.582495	No	0.55886	No	0.827879	No	0.733264
11	No	0.20303	No	0.089469	No	0.001971	No	0.017393
13	No	0.595972	No	0.072207	No	0.042873	No	0.037278
15	No	0.769439	No	0.026501	No	0.019756	No	0.020678
17	No	0.720407	No	0.092106	No	0.034111	No	0.031902
19	No	0.652024	No	0.121929	No	0.147108	No	0.251252
21	No	0.751749	No	0.499174	No	0.093399	No	0.026346
23	No	0.684518	No	0.158393	No	0.034383	No	0.028827
25	No	0.853868	No	0.651096	No	0.038439	No	0.00732
27	No	0.779986	No	0.535067	No	0.028959	No	0.004309
29	No	0.159493	No	0.719335	No	0.023159	No	0.119059
31	No	0.91702	No	0.342562	No	0.039199	No	0.004069
33	No	0.606311	No	0.345113	No	0.1277	No	0.011961
35	No	0.410562	No	0.853032	No	0.032665	No	0.079759
37	No	0.445763	No	0.797802	No	0.121882	No	0.248505
39	No	0.227193	No	0.759825	No	0.063528	No	0.420187
41	No	0.719807	No	0.317051	No	0.094788	No	0.18128
43	No	0.709899	No	0.929205	No	0.193721	No	0.340131
45	No	0.183202	No	0.652983	No	0.463794	No	0.405537
47	No	0.108852	No	0.886934	No	0.107949	No	0.971079
49	No	0.957727	No	0.55886	No	0.976299	No	0.975908
51	No	0.257453	No	0.162403	No	0.077064	No	0.542596
53	No	n/a	No	n/a	No	n/a	No	n/a
55	No	n/a	No	n/a	No	n/a	No	n/a
57	No	n/a	No	n/a	No	n/a	No	n/a
59	No	n/a	No	n/a	No	n/a	No	n/a
61	No	n/a	No	n/a	No	n/a	No	n/a
63	No	n/a	No	n/a	No	n/a	No	n/a
65	No	n/a	No	n/a	No	n/a	No	n/a
67	No	n/a	No	n/a	No	n/a	No	n/a
69	No	n/a	No	n/a	No	n/a	No	n/a

	P60							
	WT vs. *Ppt1^−/−^*		WT vs. DR-WT		WT vs. DR-*Ppt1^−/−^*		*Ppt1^−/−^* vs. *Ppt1^−/−^*	
Distance from soma (mm)	Significant?	*p*-value (FDR-corrected)	Significant?	*p*-value (FDR-corrected)	Significant?	*p*-value (FDR-corrected)	Significant?	*p*-value (FDR-corrected)
5	No	0.477723	No	0.664789	No	0.006412	No	0.020372
7	No	0.420831	No	0.714423	No	0.611685	No	0.816119
9	No	0.771676	No	0.457596	No	0.824366	No	0.984798
11	No	0.058344	No	0.136095	No	0.112691	No	0.661245
13	No	0.186948	No	0.810744	No	0.489697	No	0.935345
15	No	0.204972	No	0.894806	No	0.100379	No	0.429375
17	Yes	0.006693	No	0.878188	No	0.051701	No	0.755556
19	Yes	0.000192	No	0.96489	No	0.017115	No	0.745268
21	Yes	0.000057	No	0.60339	No	0.010933	No	0.627345
23	Yes	0.000011	No	0.099953	No	0.019504	No	0.157207
25	Yes	0.006549	No	0.219129	No	0.013333	No	0.71113
27	Yes	0.000707	No	0.13088	No	0.021889	No	0.449459
29	No	0.049382	No	0.478572	No	0.099788	No	0.974565
31	No	0.507138	No	0.822665	No	0.093195	No	0.261626
33	No	0.63427	No	0.431995	No	0.884664	No	0.566253
35	No	0.56112	No	0.800801	No	0.517757	No	0.887647
37	No	0.679379	No	0.562184	No	0.93761	No	0.679088
39	No	0.358608	No	0.568232	No	0.794186	No	0.612062
41	No	0.457378	No	0.681405	No	0.782115	No	0.356352
43	No	0.444392	No	0.83137	No	0.972842	No	0.543739
45	No	0.611317	No	0.865527	No	0.850617	No	0.865063
47	No	0.847097	No	0.366599	No	0.816893	No	0.6132
49	No	0.332585	No	0.46502	No	0.327636	No	0.714768
51	No	0.4539	No	0.42715	No	0.713331	No	0.730071
53	No	0.32822	No	0.367752	No	0.659737	No	0.185837
55	No	0.164896	No	0.944479	No	0.773024	No	0.117212
57	No	0.164896	No	0.950238	No	0.382432	n/a	n/a
59	No	0.164896	No	0.705488	No	0.382432	n/a	n/a
61	n/a	n/a	No	0.549339	n/a	n/a	n/a	n/a
63	n/a	n/a	n/a	n/a	n/a	n/a	n/a	n/a
65	n/a	n/a	n/a	n/a	n/a	n/a	n/a	n/a
67	n/a	n/a	n/a	n/a	n/a	n/a	n/a	n/a
69	n/a	n/a	n/a	n/a	n/a	n/a	n/a	n/a

	P78							
	WT vs. *Ppt1^−/−^*		WT vs. DR-WT		WT vs. DR-*Ppt1^−/−^*		*Ppt1^−/−^* vs. *Ppt1^−/−^*	
Distance from soma (mm)	Significant?	*p*-value (FDR-corrected)	Significant?	*p*-value (FDR-corrected)	Significant?	*p*-value (FDR-corrected)	Significant?	*p*-value (FDR-corrected)
5	No	0.042696	No	0.824672	Yes	0.001025	No	0.397884
7	No	0.963773	No	0.972826	Yes	0.008919	No	0.011975
9	No	0.449968	No	0.920051	No	0.124699	No	0.494187
11	No	0.124403	No	0.502538	Yes	0.000246	No	0.063402
13	Yes	0.010318	No	0.369974	Yes	0.000008	No	0.077599
15	Yes	0.006424	No	0.396186	Yes	<0.000001	No	0.031292
17	Yes	0.002359	No	0.33852	Yes	<0.000001	No	0.003346
19	Yes	0.000232	No	0.584397	Yes	<0.000001	No	0.008191
21	Yes	0.000151	No	0.257106	Yes	<0.000001	No	0.095409
23	Yes	0.000182	No	0.765976	Yes	<0.000001	No	0.006013
25	Yes	0.006791	No	0.424461	Yes	<0.000001	Yes	0.000851
27	No	0.054901	No	0.972101	Yes	0.000003	No	0.003857
29	No	0.177626	No	0.294129	Yes	0.000003	Yes	0.000265
31	No	0.340478	No	0.341287	Yes	0.000121	Yes	0.001447
33	No	0.43063	No	0.129298	Yes	0.003815	No	0.014931
35	No	0.80783	No	0.916156	No	0.079029	No	0.04226
37	No	0.54775	No	0.928594	No	0.243799	No	0.062919
39	No	0.214463	No	0.554299	No	0.180117	No	0.005381
41	No	0.019274	No	0.465019	No	0.28035	Yes	0.000484
43	No	0.028008	No	0.470463	No	0.384071	Yes	0.002221
45	No	0.026521	No	0.525234	No	0.427498	No	0.013471
47	No	0.497711	No	0.32936	No	0.34659	No	0.061376
49	No	0.511864	No	0.986315	No	0.957238	No	0.514134
51	No	0.176284	No	0.659492	No	0.479857	No	0.632634
53	No	0.717842	No	0.591459	No	0.919034	No	0.790325
55	No	0.799579	No	0.671725	No	0.39728	No	0.178828
57	No	0.39728	No	0.894499	No	0.39728	n/a	n/a
59	No	0.39728	No	0.672316	No	0.39728	n/a	n/a
61	n/a	n/a	No	0.269998	n/a	n/a	n/a	n/a
63	n/a	n/a	n/a	n/a	n/a	n/a	n/a	n/a
65	n/a	n/a	n/a	n/a	n/a	n/a	n/a	n/a
67	n/a	n/a	n/a	n/a	n/a	n/a	n/a	n/a
69	n/a	n/a	n/a	n/a	n/a	n/a	n/a	n/a

**Table 2:** Statistics for multiple *t*-tests performed in Supplementary Figure 3C.

Sholl profile in dLGN (P21–P42)		
	WT vs. *Ppt1^−/−^*	
Distance from soma (μm)	Significant?	*p*-value
5	No	0.549601
7	No	0.964156
9	No	0.668587
11	No	0.205016
13	No	0.616089
15	No	0.098918
17	No	0.062513
19	No	0.065219
21	No	0.020446
23	No	0.530833
25	No	0.926505
27	Yes	0.001545
29	No	0.01102
31	Yes	0.000034
33	Yes	0.000659
35	No	0.012683
37	No	0.042333
39	No	0.032263
41	No	0.266395
43	No	0.666866
45	No	0.816703
47	No	0.83697
49	No	0.751296
51	No	0.870362
53	No	0.859203
55	No	0.950965
57	No	0.990564
59	No	0.969813
61	No	0.969813
63	No	0.969813
65	No	0.969813
67	No	0.969813
69	No	0.969813

**Table 3:** Statistics for multiple *t*-tests performed in Figure 6D.

Sholl profile data Figure 6D statistics (*t*-tests)		
	Vehicle vs. FK506 (*Ppt1^−/−^*)	
Distance from soma (μm)	Significant?	p-value
5	No	0.7207
7	No	0.3281
9	No	0.0683
11	No	0.1571
13	No	0.6747
15	No	0.5308
17	No	0.856
19	No	0.1679
21	No	0.0306
23	Yes	0.036
25	Yes	0.5008
27	No	0.0018
29	Yes	0.006
31	Yes	0.0018
33	Yes	0.0042
35	Yes	0.0012
37	Yes	0.0135
39	Yes	0.0852
41	No	0.2468
43	No	0.3437
45	No	0.4559
47	No	0.6262
49	No	0.6153
51	No	0.8887
53	No	0.852
55	No	>0.9999
57	No	>0.9999
59	No	>0.9999
61	No	>0.9999
63	No	>0.9999
65	No	>0.9999
67	No	>0.9999
69	No	0.7207

**Table 4:** Key resources table.

Reagent type (species) or resource	Designation	Source or reference	Identifiers	Additional information (antibody dilution)
Strain, strain background (*Mus musculus*)	B6;129-Ppt1^tm1Hof^/J	Jax stock #: 004313	Gupta, PNAS, 2001; RRID: MGI:004313	
Antibody	Rabbit polyclonal anti-Akap150	Gift from Mark Dell’Acqua	RRID: AB_2532138	1:1,000 (Brandao et al., 2012)
Antibody	Mouse monoclonal anti-GluA1	Millipore Sigma	Catalog #MAB2263; RRID: AB_11212678	1:1,000 dilution
Antibody	Mouse monoclonal anti-GluN2B	UC Davis/NIH NeuroMab Facility	Cat: 75/097; RRID: AB_10673405	1:1,000
Antibody	Mouse monoclonal anti-β-actin	Thermo Fisher Scientific	Cat: A2228; RRID: AB_476697	1:2,000
Antibody	Donkey anti-Rabbit IgG Secondary Antibody, HRP	Thermo Fisher Scientific	Cat: SA1-200; RRID: AB_325994	1:5,000 or 1:10,000
Antibody	Peroxidase AffiniPure Goat Anti-Mouse IgG	Jackson ImmunoResearch	Cat: 115–035–146; RRID_AB_2307392	1:1,000 for biotinylation; 1:5,000 or 1:1,0000 for all else
Antibody	GFP Polyclonal Antibody, Alexa Fluor 488	Thermo Fisher Scientific	Cat: A-21311; RRID: AB_221477	1:1,000
Antibody	Rabbit polyclonal anti-Iba1	Fujifilm/Wako	Cat: 019–1974; RRID: AB_839504	1:1,000
Antibody	Gt anti-rabbit 633	Thermo Fisher Scientific	Cat: A-21070; AB_2535731	1:400
Antibody	Gt anti-mouse 488	Thermo Fisher Scientific	Cat: A28175; RRID: AB_2536161	1:400
Commercial assay or kit	Dynabeads™ M-280 Streptavidin	Thermo Fisher Scientific	Cat: 11205D	
Recombinant DNA reagent	GFP-mNFATc3	Gift from Mark Dell’Acqua		Mouse NFATc3 with GFP tag
Recombinant DNA reagent	CAG-mCherry			
Chemical compound, drug	Tetrodotoxin-citrate (TTX)	Tocris	Cat: 1069	Treatment: 1 μM
Chemical compound, drug	NASPM	Tocris	Cat: 2766	Treatment: 10 μM
Chemical compound, drug	5KDa maleimide PEG (for APEGS)	NOF America Corporation	Cat: ME-050MA	
Chemical compound, drug	Cholera toxin subunit B 488	Thermo Fisher Scientific	Cat: C34775	1 mg/mL in PBS
Chemical compound, drug	Cholera toxin subunit B 555	Thermo Fisher Scientific	Cat: C34776	1 mg/mL in PBS
Software, algorithm	Fiji			
Software, algorithm	Prism 9.0.1	GraphPad		

### 2.3 Transcardial perfusion and AFSM quantification

Wild-type and *Ppt1^−/−^* mice were anesthetized using isoflurane and transcardially perfused with ice-cold PBS (pH 7.4, ~30 mL/mouse), followed by 4% paraformaldehyde (PFA) in PBS (~15 mL/mouse). Brains were removed and post-fixed overnight at 4°C in 4% PFA and transferred to PBS, pH 7.4, containing 30% sucrose solution for 48 h prior to sectioning at either 50 or 100 μm in cold PBS using a Vibratome 1,000 (Technical Products International, St. Louis, MO). Serial sections were stored free floating in cryoprotectant solution (30% glycerol, 30% ethylene glycol in PBS) at −20°C until analysis of AFSM or immunohistochemistry was performed.

For AFSM analysis (as in Koster et al., 2019), 3–4 mid-sagittal sections were mounted on Superfrost Plus microscope slides (VWR) using Vectamount mounting media containing DAPI (Vector Laboratories). Images were acquired for at least two sections from each animal using a Zeiss LSM710 confocal laser scanning microscope at 40× magnification. DAPI signal was visualized using excitation at 405 nm, while AFSM was detected using 561 nm excitation. Although AFSM signal is detectable without any deliberate staining across a wide spectral range, we chose to quantify it in the red fluorescence range to allow for consistency across experiments where the green (488 nm) channel was occupied by immunostaining for another protein of interest (e.g., Iba1). All sections were imaged using identical capture conditions. Quantification of AFSM was performed by generating a binary mask of AFSM-positive pixels (i.e., fluorescent signal in the 561 nm channel) in FIJI. An identical threshold was applied to each image. Percent area occupied by AFSM puncta that satisfied the threshold was then calculated using the “analyze particles” tool in FIJI. This analysis was performed for 2–4 sections (a total of 10–20 images, as imaging an entire cortical column is typically five interlaced images) from each animal and averaged together to give a single value, representative of the total area occupied by AFSM in the cortical column imaged. Animal numbers are reported in the relevant figure legends.

### 2.4 Cortical thickness measurement

Low magnification (4×) images were acquired from 2 to 3 matched sections for all animals used for AFSM analysis using a Zeiss Axio Imager M1. Images were analyzed in Fiji by drawing an ROI perpendicular to the cortical surface that extended down to the start of the subcortical white matter, i.e., at the lowest bounding edge of cortical L6, where DAPI staining sharply disappears. ROI length was measured for three mid-sagittal sections from each animal corresponding to V1. These values were averaged to give the cortical thickness in mm.

### 2.5 Immunohistochemistry and microglial morphology analysis

For immunohistochemistry, 3–4 medial sections were first incubated in TBS for 10 min before undergoing permeabilization (TBS + 0.5% Triton X-100) for 30 min at RT. Next, samples underwent antigen retrieval by heating in tris-EDTA (pH 9.0) at 95°C for 30 min before being equilibrated to room temperature in Tris-EDTA solution for 40 min. Tissue was then blocked (TBS + 0.1% Triton X-100, 4% BSA, and 5% normal goat serum) for 2 h at RT before being incubated in rabbit anti-Iba1 (1:1,000, in TBS + 0.1% Triton X-100 + 2% BSA) for 48 h. After washing four times, 10 min each, tissue was incubated in Alexa Fluor 488 goat anti-rabbit (1:1,000; Thermo Fisher) overnight at RT. The tissue was then washed (4×, 10 min) and mounted as above.

Microglial images were acquired with a Zeiss LSM710 confocal microscope either at 10× (low magnification images) or 63× for Sholl analysis. 63× images were acquired as 30–60 plane *Z*-stacks (*Z*-interval = 2 μm) in random fashion (the only criterion is that at least one full microglia had to be centered in the stack) at the border of layers 2/3 and 4 in the visual cortex. 3–4 images were taken from two tissue sections for each animal. Images were analyzed using the Sholl analysis tool in Fiji by lab members blinded to the condition. Briefly, the image was collapsed into a maximum-intensity projection to ensure all microglia processes were captured, and individual microglia were outlined with a freehand ROI. The surrounding area was removed (“clear outside” function in Fiji), and the image was then thresholded to generate a mask of all microglia processes, and an ROI was created at the center of the cell soma. The mask containing one individual microglia was then skeletonized using the “skeletonize” plugin in Fiji. Sholl analysis was performed according to these parameters using the Fiji Sholl tool: start radius (from the center of cell soma ROI) = 5 μm, step size = 2 μm, end radius = 70 μm. The number of intersections at each 2 μm step was averaged for all microglia from each animal and counted as one *n*.

To analyze the number of microglia across age, genotype, and condition (LR vs. DR), the number of Iba1-positive cell bodies was counted manually in a 0.135 × 0.135 × 0.05 mm (width × length × depth) volume using the imaging parameters described above. Cell bodies straddling the top and right borders of the image were not counted, while those on the left and bottom borders were. The number of microglia in this view field was then extrapolated to estimate the number of microglia in a 1 mm^3^ volume.

### 2.6 Cholera toxin B subunit injection

Mice between P20 and P40 were anesthetized via isoflurane inhalation (4% induction, 1–1.5% maintenance) and placed in a stereotaxic frame (RWD Life Science Inc.). After expressing a small amount of vitreous from the eye, intravitreous injections of 2 μL fluorophore-conjugated CTB (1 mg/mL; 488 nm conjugate in the left eye, 555 nm conjugate in the right eye) were performed for each mouse. Following the injection, antibiotic ointment was applied to each eye, and the animals were allowed to recover in their home cage for 24 h before undergoing transcardial perfusion as detailed above. Sectioned brains (100 μm sequential coronal sections encompassing the entire dLGN) were then assessed for the quality of the injections by a blinded researcher, and only those brains with well-traced retinogeniculate projections were immunostained for Iba1. Matched sections were then imaged under high magnification at the border of converging retinal projections from both eyes in each dLGN using an LSM710 confocal microscope and compared for microglial morphology (Sholl analysis) as described above.

### 2.7 Acyl-biotin exchange (ABE) assay for palmitoyl-proteomics

WT and *Ppt1^−/−^* occipital cortices from animals aged to P42 were used for palmitoyl-proteomic analysis. Lysates and synaptosomes were collected as described above. The palmitoyl-proteomic protocol was then carried out according to Wan et al. (2007) with slight modifications. Before beginning the assay, a BCA assay was performed to start with equal (600 μg) protein content for each sample. Blocking (N-ethylmaleimide; NEM), hydroxylamine, and biotinylation (HPDP-biotin) steps were all performed as recommended in the protocol. The elution protocol was also followed (Wan et al., 2007), with the exception that instead of streptavidin resin, magnetic streptavidin-coated beads (Dynabeads™, Thermo Fisher) were used (100 μL beads/sample). The final eluent was frozen at −80°C and prepared for mass spectrometry (see below). Due to the small starting material (occipital cortex only), the whole procedure was scaled down into 2 mL tubes, including chloroform-methanol precipitations, which were carried out with the following volumes: 150 μL sample, 600 μL methanol, 150 μL chloroform, and 450 μL water. This limited protein loss was evident in trial runs using 15 mL conical tubes.

### 2.8 Mass spectrometry

Eluents from the ABE assay were dried for approximately 30 min and digested using the S-trap Micro Spin Column Digestion Protocol (Protifi, Huntington, NY) with minor changes. Briefly, 30 μL of 10% sodium dodecyl sulfate (SDS) and 100 mM triethylammonium bicarbonate (TEAB) with Pierce protease inhibitor cocktail (Thermo Fisher Scientific, Waltham, MA) and phosphatase inhibitors (10 mM sodium pyrophosphate, 1 mM PMSF, 1 mM sodium orthovanidate, and 1 mM β-glycerolphosphate). Proteins were reduced with a final concentration of 20 mM dithiothreitol (DDT) at 95°C for 10 min, followed by alkylation in the dark at room temperature with 40 mM of iodoacetamide. Next, phosphoric acid was added for a final concentration of 1.2%. Samples were briefly vortexed to mix before 300 μL of S-trap binding buffer (90% MeOH, 100 mM TEAB) was added. Samples were vortexed again prior to loading onto the S-Trap Micro Spin Columns. After four washes with 150 μL of S-trap binding buffer with centrifugation at 1,000×*g*, 40 μL of 50 mM TEAB containing 0.75 μg of trypsin was added and incubated overnight at 37°C.

Peptides were eluted with 40 μL of each of the following solutions: 50 mM TEAB, 0.2% formic acid (FA), 50% acetonitrile (ACN), and 0.1% FA. The spin column was spun at 4,000×*g* after adding each solution. Pooled eluents were dried down prior to resuspension in 100 μL of 3% ACN and 0.1% FA.

#### 2.8.1 LC–MS analysis

Three microliters of resuspended samples were injected for LC–MS/MS analysis, similar to a previously mentioned method (Nguyen et al., 2019). Briefly, peptides were loaded onto a Thermo NanoViper trap column (75 μm × 20 mm, 3 μm C18, 100 Å) (Thermo Fisher Scientific, Bremen, Germany) using an Agilent 1,260 Infinity nanoLC system (Agilent Technologies, Santa Clara, CA) and washed for 10 min with 0.1% FA at 2 μL/min. Peptides were separated with a 120-min gradient (from 5 to 60% ACN with 0.1% FA), at 0.25 μL/min flow rate, on an Agilent Zorbax 300SB-C18 column (75 μm × 150 mm, 3.5 μm, 300 Å). Data were collected using data-dependent acquisition (DDA) analysis by a Thermo Q Exactive mass spectrometer (Thermo Fisher Scientific, Bremen, Germany). Settings for the mass spectrometer are as follows: capillary temperature at 250°C, spray voltage 1.5 kV, MS1 scan at 70,000 resolution, scanning from 375 to 1,600 m/z, automatic gain control (AGC) target 1E6 for a maximum injection time (IT) of 100 ms. The 10 most abundant peaks within an MS1 spectrum were isolated for MS/MS, with an isolation width of 1.5 m/z and a dynamic active exclusion set for 20 s. MS/MS spectra were collected at 17,500 resolution for a maximum of 50 ms or a minimum of 1E5 ions. Normalized collision energy (NCE) was set at 27%. Masses with charges of 1 and larger than 6 were excluded from MS/MS analysis.

### 2.9 Analysis for palmitoyl-proteomics

Raw files were searched with Proteome Discoverer 2.3 (Thermo Fisher Scientific, Waltham, MA) using the Sequest HT search engine against the UniProt *Mus musculus* database (22,286 gene sequences; downloaded on 27 April 2017). Mass error tolerance was set to 10 ppm for precursors, cleaved by trypsin, allowing a maximum of two missed cleavages, with sequence lengths between 6 and 144 amino acids. Fragment masses were searched with a tolerance of ±0.02 Da. Dynamic modifications included oxidation (M), deamidation (N, R, Q), and acetylation (N-terminus). Carbamidomethylation was set as a static modification (C). Both peptides and PSMs were set to a target false discovery rate (FDR) ≤0.01 for matches with high confidence. Label-free quantification (LFQ) was performed using precursor ion intensity. Samples were normalized using the average intensity of all peptides. The top five most abundant peptides were used for protein abundance calculation. *t*-test was used to determine the *p*-values between the two conditions.

Datasets filtered for two unique peptides were further narrowed by filtering for proteins showing an increase in their abundance ratio (*Ppt1^−/−^/*WT) of >1.2-fold. These filtered gene lists for lysates and synaptosomes were input separately into the SynGO online tool (Koopmans et al., 2019). The genes encompassed in the top significant biological process (BP) SynGO term for lysates and synaptosomes were then input into STRING, the online protein–protein interaction database.^1^ A K-means cluster analysis was performed to detect clusters of functionally related proteins and for clarity of visualization.

### 2.10 APEGS assay from visual cortices

The APEGS assay was performed as described (Kanadome et al., 2019), following the guidelines for tissue samples. Homogenate buffer as described in the APEGS protocol (20 mM Tris–HCl, 2 mM EDTA, 0.32 M sucrose, pH 8.0) was used to homogenize WT and *Ppt1^−/−^* occipital cortices. Lysates and synaptosomes were then brought to 300 μg total protein in a final volume of 0.5 mL buffer B (PBS containing 4% SDS, 5 mM EDTA, 8.9 M urea, and protease inhibitors). Proteins were then reduced by the addition of 25 mM Bond-Breaker™ TCEP (0.5 M stock solution, Thermo Fisher) and incubation at RT for 1 h. To block free thiols, freshly prepared N-ethylmaleimide (NEM) in 100% ethanol was added to lysates (to 50 mM), and the mixture was rotated end-over-end for 3 h at RT. Following 2× chloroform-methanol precipitation, samples were divided into +hydroxylamine (HA) and −HA groups, which were exposed to three volumes of HA-containing buffer (1 M HA to expose palmitoylated cysteine residues) or tris-buffer control (−HA), respectively, for 1 h at 37°C. Following chloroform-methanol precipitation, the samples were solubilized and exposed to 10 mM TCEP and 20 mM mPEG-5 k (Laysan Bio Inc., see Table 4) for 1 h at RT with shaking (thereby replacing palmitic acid with mPEG-5K on exposed cysteine residues). Following the final chloroform-methanol precipitation, samples were solubilized in 70 μL of PBS containing 1% SDS, and protein concentration was measured by BCA assay (Pierce). Samples were then brought to 20 μg protein in Laemmli buffer with 2% β-mercaptoethanol for immunoblot analyses as above. Quantification of palmitoylated vs. non-palmitoylated protein was carried out for standard immunoblot analysis, with the additional consideration that signal from palmitoylated bands demonstrating the APEGS-dependent molecular weight shift was divided by the signal from the non-palmitoylated band, the location of which was verified by matching to the −HA control sample. This ratio was divided by β-actin control from the same lane for normalization.

### 2.11 Primary cortical neuron culture

For primary cortical neuron cultures, embryos at embryonic day 15.5 from timed-pregnant *Ppt1^−/+^* dams were removed, decapitated under anesthesia, and cortices resected. All dissection steps were performed in ice-cold HBSS, pH 7.4. Following cortical resection, tissue from each embryo was individually collected in a separate microtube, genotyped, and digested in HBSS containing 20 U/mL papain and DNAse at 37°C (20 min total; tubes flicked at 10 min) before sequential trituration with 1 mL (~15 strokes) and 200 μL (~10 strokes) pipettes, generating a single-cell suspension. For live-cell/immunohistochemical experiments, cells were counted and then plated at 150,000–180,000 cells/well in 24-well plates containing poly-d-lysine/laminin-coated coverslips. Plated cells were incubated at 37°C in plating medium (neurobasal medium containing B27 supplement, l-glutamine, and glutamate) for 3–5 DIV, before replacing half medium every 3 days with feeding medium (plating medium without glutamate). For synaptic scaling experiments, neurons were treated with either bicuculline (20 mM, solubilized in DMSO, Tocris) or TTX citrate (1 mM, solubilized in sterile water, Tocris) for 48 h where indicated.

### 2.12 GFP-NFAT nuclear translocation analyses

Analysis of NFAT nuclear translocation following culture-wide depolarization was performed as in Murphy et al. (2014) with minor modification. Neurons were transfected at DIV 12 with a 1:1 mixture of CAG-mCherry and mouse GFP-NFATc3 (pCMV-SGFP2-mNFATc3.dna, courteously provided by Dr. Dell’Acqua), 1 mg DNA per coverslip using Lipofectamine^®^ 2000 (Thermo Fisher) as above. A subset of neurons was treated with TTX (1 μM) for 48 h leading up to the assay to induce synaptic scaling. Solutions were composed as follows: Tyrode + TTX (in mM: 135 NaCl, 5 KCl, 2 CaCl_2_, 1 MgCl2, 25 HEPES, and 10 glucose, pH 7.4 and 1 mM TTX), depolarization solution was isotonic, but with 50 mM KCl (85 mM NaCl) and without TTX, and recovery solution was standard Tyrode without TTX. Cells were incubated at 37°C in between steps. For every coverslip of depolarized (KCl) neurons, a control with no depolarization (sham, Tyrode solution + TTX) and depolarization with the selective GluA1-containing AMPAR blocker NASPM (KCl + 10 μM NASPM) added were performed in parallel. Following fixation at the end of the assay, cells were immunostained with anti-GFP 488 antibody (Thermo Fisher) following the protocol above to amplify the GFP-NFAT signal. GFP-NFAT nucleus/soma ratio was analyzed by manually tracing the nucleus, based on the DAPI staining signal, and soma as independent ROIs and dividing the integrated fluorescence value (measured by multi-measure tool in Fiji) for the nucleus by that for the soma.

### 2.13 FK506 dosing

Animals were treated with FK506 at a dose of 3 mg/kg/day (Muthuraman and Sood, 2010), similar to a previous study (Yoshiyama et al., 2007). Specifically, FK506 (Selleck Chemicals) was solubilized in DMSO at a concentration of 9 mg/mL to make stock solution aliquots. The FK506 stock was solubilized into sucrose-supplemented (2%) drinking water in mice at a dilution of 1:400 (bringing the final DMSO concentration to 0.25%) and changed every other day. Considering animals drink ~4 mL/day, they are expected to consume 0.09 mg/day via this passive administration route, or the equivalent of 3 mg/kg at an average weight of 30 g. Analysis of AFSM and gliosis in FK506-treated animals was performed as mentioned above.

### 2.14 Motor assessment (Rotarod) of vehicle- and FK506-treated *Ppt1^−/−^* mice

Each animal performed three trials on a fixed speed rotarod, and the latency to fall was timed for each animal manually by an observer. The average latency to fall was calculated for each subject.

## 3 Results

### 3.1 Palmitoyl-proteomics points to over-palmitoylation of Akap5 in *Ppt1^−/−^* visual cortex

A limitation of our previous studies demonstrating that loss of Ppt1 function impairs the function and plasticity of synaptic receptors (Koster et al., 2019, 2023) is that it remains largely unclear how these early-disease changes contribute to classical disease features, like AFSM accumulation or neuroinflammation. To uncover dysregulated molecular pathways in CLN1 that might provide a link between these processes, we performed a palmitoyl-proteomic screen using the acyl-biotin exchange method (Drisdel and Green, 2004; Roth et al., 2006; Wan et al., 2007) on visual cortical lysates and synaptosomes from young (postnatal day (P) 42) *Ppt1^−/−^* mice. We chose mice at this timepoint to capture early changes to synaptic protein palmitoylation, prior to the onset of severe disease.

**Figure 1:**
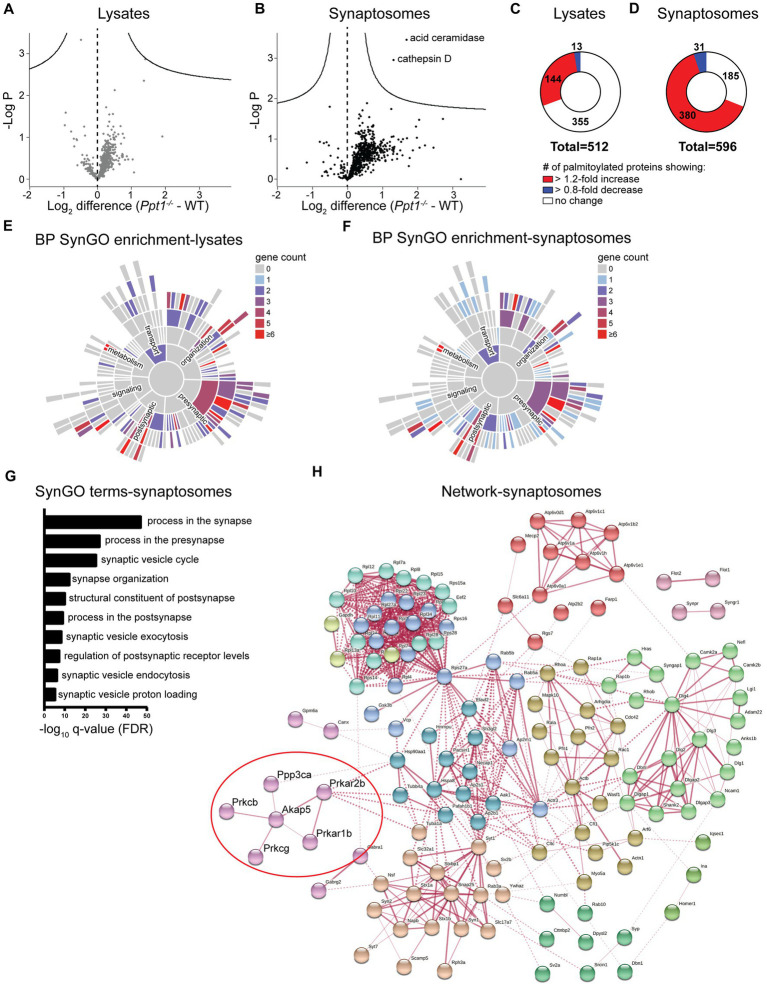
Palmitoyl-proteomics points to excessive palmitoylation of Akap5 and associated signaling proteins in the *Ppt1^−/−^* visual cortex. **(A)** Volcano plot showing the log2 fold change in palmitoyl-protein expression from lysates of WT and *Ppt1^−/−^* visual cortex at P42. *N* = 6 visual cortices/group. **(B)** Volcano plot showing the log2 fold change in palmitoyl-protein expression from synaptosomes of WT and *Ppt1^−/−^* visual cortex. *N* = 6 visual cortices/group. **(C)** Breakdown of the proportion of proteins exhibiting a 1.2-fold or greater (red), 0.8-fold or lower (blue), or no change (white) in the abundance ratio *Ppt1^−/−^*/WT from visual cortical lysates. **(D)** Breakdown of the proportion of proteins exhibiting a 1.2-fold or greater (red), 0.8-fold or lower (blue), or no change (white) in the abundance ratio *Ppt1^−/−^*/WT from visual cortical synaptosomes. **(E)** SynGO annotation of the palmitoyl-proteome of visual cortical lysates from WT and *Ppt1^−/−^* mice. BP, biological process. **(F)** SynGO annotation of the palmitoyl-proteome of visual cortical synaptosomes from WT and *Ppt1^−/−^* mice. **(G)** Top 10 enriched SynGO terms from proteins increased 1.2-fold in *Ppt1^−/−^* visual cortical synaptosomes. **(H)** Network analysis of the genes increased in *Ppt1^−/−^* synaptosomes by 1.2-fold that were annotated with the top biological process SynGO term “process at the synapse.” Red circle denotes Akap5 and associated signaling protein subunits.

This analysis detected 512 palmitoylated proteins in lysates and 596 in synaptosomes from WT and *Ppt1^−/−^* visual cortices (Figures 1A,B). In lysates, we did not detect any significant changes in the abundance ratio (*Ppt1^−/−^*/WT) for any proteins (Figure 1A). In synaptosomes, we detected significantly increased palmitoylation levels of only two proteins, acid ceramidase and cathepsin D, demonstrating that these proteins are consistently overrepresented in the CLN1 brain across several studies (Figure 1B; Chandra et al., 2015; Sleat et al., 2017; Atiskova et al., 2019; Gorenberg et al., 2022). Despite only a few proteins showing statistically significant excessive palmitoylation, we found that most of the identified proteins showed modest over-palmitoylation in the *Ppt1^−/−^* brain, particularly in synaptosomes (Figures 1A,B). Specifically, in *Ppt1^−/−^* lysates, 144 proteins (28.1%) show a > 1.2-fold increase in the raw abundance ratio compared to WT, while only 13 (2.5%) show a < 0.80-fold change (Figure 1C). This trend is even more robust in *Ppt1^−/−^* synaptosomes, with 380 proteins (63.8%) demonstrating a > 1.2-fold change increase, and only 31 (5.2%) showing a < 0.80-fold change reduction (Figure 1D). These data indicate a bulk increase in the palmitoylation level of synaptic proteins in young mice with loss of Ppt1 function, consistent with Ppt1 regulating a substantial proportion of the synaptic palmitome (Gorenberg et al., 2022).

To identify the characteristics of synaptic proteins that are overrepresented in the palmitoyl fraction of *Ppt1^−/−^* visual cortices, we performed a synapse-specific gene ontology analysis using SynGO (Koopmans et al., 2019). For both datasets, SynGO analysis demonstrated a robust enrichment for synaptic proteins compared to a whole-brain proteomic background dataset (Figures 1E,F), emphasizing the role of palmitoylation at the synapse (Sanders et al., 2015). The top 10 enriched SynGO terms demonstrated substantial overlap between lysates and synaptosomes, which is expected given the enrichment for palmitoylated proteins in both populations. “Process in the synapse,” “process in the presynapse,” “structural constituent of the postsynapse,” “process in the postsynapse,” and “regulation of postsynaptic receptor levels” are represented in the top 10 enriched terms in both lysates and synaptosomes (Figure 1G and Supplementary Figure 1A).

To highlight specific pathways that are dysregulated by a lack of Ppt1, we performed a network analysis on all genes annotated with the top SynGO term “process in the synapse” using the online protein–protein interaction database, STRING (Snel et al., 2000; Szklarczyk et al., 2017). As expected from the SynGO analysis, the networks in lysates and synaptosomes demonstrated substantial overlap (Figure 1H and Supplementary Figure 1B). We took note of a particular cluster that appeared with minor variation in both lysates and synaptosomes, consisting of *Akap5*, the cAMP-dependent protein kinase subunits *Prkacb*, *Prkar1b*, and *Prkar2b*, the protein kinase C subunits *Prkcb* and *Prkcg*, and the calcineurin subunit *Ppp3ca* (Figure 1H, red circle).

The *Akap5* gene encodes the A-kinase anchoring protein 5 (Akap5), a postsynaptic scaffolding protein that anchors protein kinase A, protein kinase C, and calcineurin to the postsynaptic density, where it indirectly interacts with AMPARs (Colledge et al., 2000; Robertson et al., 2009). Through these interactions, Akap5 can link synaptic activity to downstream signaling (Sanderson and Dell’Acqua, 2011), making it a top candidate for a potential link between aberrant synaptic plasticity and long-term pathogenic changes to the *Ppt1^−/−^* brain. This notion is further supported by two related lines of evidence. First, that Akap5 regulates calcium-permeable (CP)-AMPAR incorporation during synaptic scaling (Diering et al., 2014; Sanderson et al., 2018), and second, our recent finding that synaptic upscaling is affected by loss of Ppt1 function, particularly *in vitro* (Koster et al., 2023). Therefore, we focused on whether Akap5 levels are dysregulated in the *Ppt1^−/−^* brain.

### 3.2 Increased palmitoylation of Akap5 in *Ppt1^−/−^* synaptosomes

**Figure 2:**
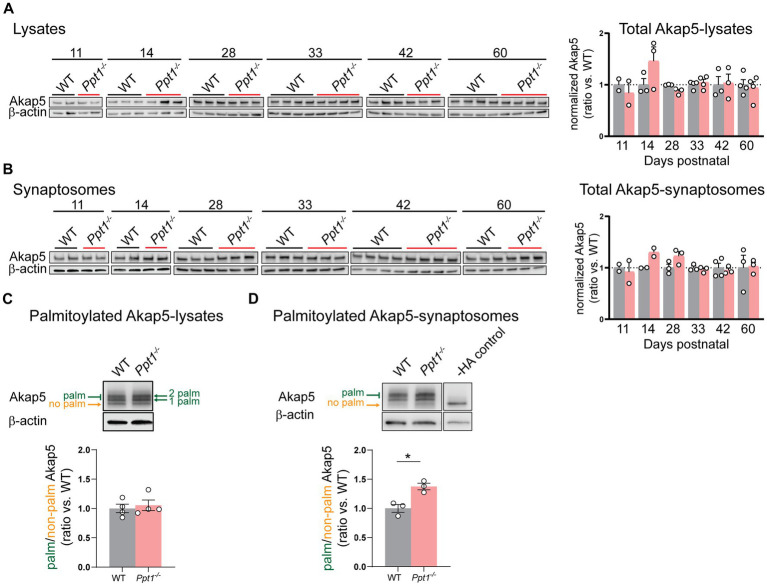
AKAP palmitoylation is increased in *Ppt1^−/−^* visual cortical synaptosomes. **(A)** Representative immunoblots (left) and quantification (right) of Akap5 levels in visual cortical lysates across ages P11–P60. **(B)** Representative immunoblots (left) and quantification (right) of Akap5 levels in visual cortical synaptosomes across ages P11–P60. **(C)** Representative immunoblot and quantification of APEGS-processed visual cortical lysates from WT and *Ppt1^−/−^* mice probing for Akap5 at P42. *N* = 3–4 mice/group. **(D)** Representative immunoblot and quantification of APEGS-processed visual cortical synaptosomes from WT and *Ppt1^−/−^* mice probing for Akap5 at P42. The −HA control shows the molecular weight of the non-palmitoylated Akap5 species. *t*-test: *p = 0.0120. *N* = 3 mice/group. Note that, unfortunately, one set of synaptosome samples was lost during APEGS processing during chloroform-methanol precipitation.

We first measured Akap5 levels across cortical development (P11–P60) and did not detect changes in the total levels of Akap5 in lysates or synaptosomes of *Ppt1^−/−^* visual cortices (Figures 2A,B). Furthermore, we did not detect increased Akap5 palmitoylation in visual cortical lysates at P42 using the acyl-PEGyl exchange gel shift (APEGS) assay (Figure 2C; Yokoi et al., 2016; Kanadome et al., 2019). However, performing the APEGS assay in visual cortical synaptosomes revealed that Akap5 is excessively palmitoylated at *Ppt1^−/−^* synapses at this age (Figure 2D). Extending our previous *in vitro* observations (Koster et al., 2019), we also demonstrated increased GluN2B palmitoylation in *Ppt1^−/−^* visual cortical synaptosomes (Supplementary Figure 2).

### 3.3 Induction of synaptic upscaling *in vivo* causes excessive upregulation of synaptic GluA1 in *Ppt1^−/−^* mice

As mentioned, converging lines of evidence highlight the centrality of Akap5 and its associated signaling molecules in regulating synaptic scaling (Diering et al., 2014; Sanderson et al., 2018). Consistent with these accumulating data, we demonstrate here an over-palmitoylation of Akap5 in *Ppt1^−/−^* brains and have shown previously that loss of Ppt1 leads to exaggerated synaptic upscaling of CP-AMPARs. However, the bulk of our prior synaptic upscaling experiments were performed in cortical neurons (Koster et al., 2023). Therefore, to test if this plasticity mechanism was also affected by loss of Ppt1 function *in vivo*, we employed a dark rearing (dark rearing/dark reared, DR) protocol in WT and *Ppt1^−/−^* mice that reliably induces upscaling in the visual cortex (Goel et al., 2006, 2011; Goel and Lee, 2007).

**Figure 3:**
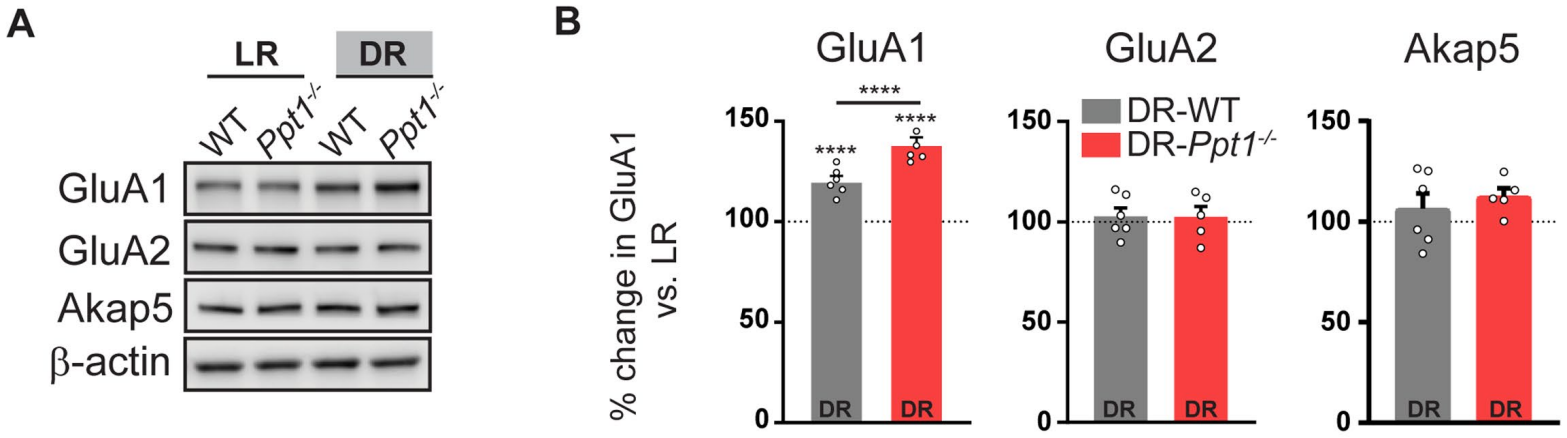
Induction of synaptic upscaling *in vivo* causes excessive upregulation of synaptic GluA1 in *Ppt1^−/−^* mice. **(A)** Representative immunoblots and **(B)** quantification of GluA1, GluA2, and Akap5 levels in LR-WT, LR-*Ppt1^−/−^*, DR-WT, and DR-*Ppt1^−/−^* visual cortical synaptosomes at P42. *N* = 5–6 animals/group. Two-way ANOVA for GluA1: interaction genotype × rearing condition (*F*(1, 20) = 17.00, ***p = 0.0005); main effect of genotype (*F*(1, 20) = 17.00, ***p = 0.0005); main effect of rearing condition (*F*(1,20) = 172.6, *p* < 0.0001). Tukey’s multiple comparison indicated on the graph: ****p < 0.0001 LR-WT vs. DR-WT; ****p < 0.0001 LR-*Ppt1^−/−^* vs. DR-*Ppt1^−/−^*; ****p < 0.0001 DR-WT vs. DR-*Ppt1^−/−^*. Data represent mean ± SEM.

Corroborating previous findings (Goel et al., 2006, 2011; Goel and Lee, 2007; Diering et al., 2014), levels of synaptosomal GluA1, but not GluA2 or Akap5, were increased in both DR-WT and DR-*Ppt1^−/−^* visual cortices compared to their light reared (light rearing/light reared, LR) counterparts (Figures 3A,B). Notably, the increase of GluA1 levels in DR-*Ppt1^−/−^* cortices exceeded those in DR-WT animals (Figures 3A,B), indicating synaptic upscaling of GluA1-containing AMPARs is exaggerated *in vivo* as well as *in vitro* (Koster et al., 2023).

### 3.4 Nuclear factor of activated T cells (NFAT) nuclear translocation is increased in upscaled *Ppt1^−/−^* neurons

Upon synaptic calcium influx, like that from CP-AMPARs, activation of Akap5-associated calcineurin leads to dephosphorylation of the nuclear factor of activated T cells (NFAT) (Sanderson and Dell’Acqua, 2011; Li et al., 2012; Murphy et al., 2019). In turn, NFAT undergoes nuclear translocation and regulates transcription (Pan et al., 2012). Therefore, we hypothesized that an overload of palmitoylated Akap5 and excessive CP-AMPAR upregulation leads to the NFAT pathway being sensitized in upscaled *Ppt1^−/−^* neurons.

**Figure 4:**
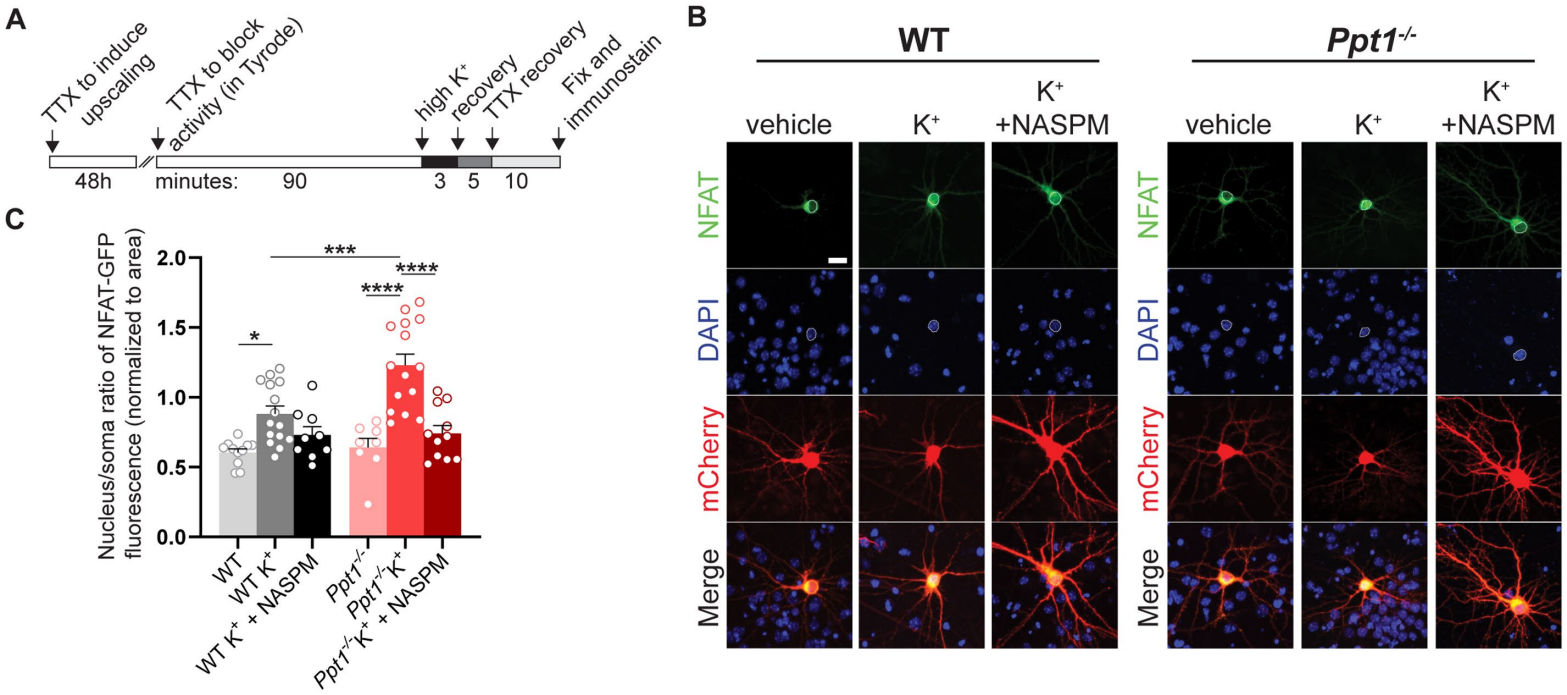
Sensitization of NFAT nuclear translocation in upscaled *Ppt1^−/−^* neurons. **(A)** Schematic representation of the experimental design for depolarization (K^+^) induction of NFAT activity (nuclear translocation), as previously described (Murphy et al., 2014). **(B)** Representative images of NFAT localization from WT and *Ppt1^−/−^* neurons in response to either sham or K^+^-induced depolarization. The outline of the nucleus (saturated DAPI signal) is drawn in each image. Scale bar = 20 μm. **(C)** Quantification of the nucleus/soma ratio of NFAT fluorescence in WT and *Ppt1^−/−^* upscaled neurons in sham depolarization, K^+^ depolarization, and K^+^ depolarization + NASPM (10 μM) conditions. Two-way ANOVA: interaction genotype × treatment (*F*(2, 62) = 4.675, *p = 0.0129); main effect of genotype (*F*(1, 62) = 5.882, *p = 0.0182); main effect of treatment (*F*(2, 62) = 26.23, ****p < 0.0001). Tukey’s multiple comparison indicated on the graph: *p = 0.0245 WT vs. WT K^+^; ****p < 0.0001 *Ppt1^−/−^* vs. *Ppt1^−/−^* K^+^; ****p < 0.0001 *Ppt1^−/−^* K^+^ vs. *Ppt1^−/−^* K^+^ + NASPM; ***p = 0.006 WT K^+^ vs. *Ppt1^−/−^* K^+^.

To test this hypothesis, we performed a nuclear translocation assay as described in Murphy et al. (2014), following 48-h pretreatment of TTX to induce upscaling in WT and *Ppt1^−/−^* neurons (Figure 4A). Following synaptic upscaling, WT and *Ppt1^−/−^* neurons were depolarized (acute incubation in isotonic high K^+^ solution) and fixed for assessment of NFAT [specifically, the NFATc3 isoform (Murphy et al., 2014)] nuclear translocation (Figures 4B,C). While high K^+^-induced depolarization triggered the nuclear translocation of NFAT-GFP in both WT and *Ppt1^−/−^* neurons, the nuclear/soma ratio was significantly higher in *Ppt1^−/−^* cells, indicating a greater responsiveness to depolarization (Figures 4B,C). To test whether this effect resulted from an increased contribution of CP-AMPARs in upscaled *Ppt1^−/−^* neurons, we performed the same assay and treated a subset of neurons with NASPM (10 μM), the CP-AMPAR selective antagonist (Koike et al., 1997), during the depolarization period. We found that NASPM reduced NFAT nuclear translocation to a greater degree in *Ppt1^−/−^* cells compared to WT (Figures 4B,C). Together, these data suggest that calcium influx through CP-AMPARs more robustly drives downstream signaling through calcineurin and NFAT, which are both anchored by Akap5, in upscaled *Ppt1^−/−^* neurons.

### 3.5 Excessive synaptic upscaling is associated with accelerated neuroinflammation in *Ppt1^−/−^* visual cortex

Although the panel of NFAT-target transcripts is incompletely characterized in neurons (Jayanthi et al., 2005; Luoma and Zirpel, 2008; Vashishta et al., 2009; Gómez-Sintes and Lucas, 2010), NFAT activation is associated with proinflammatory signaling in immune cell types, including microglia (Nagamoto-Combs and Combs, 2010; Minematsu et al., 2011; Pan et al., 2012). Neuroinflammation is a key correlate of disease severity in CLN1 and *Ppt1^−/−^* mice (Kielar et al., 2007; Palmer et al., 2013; Macauley et al., 2014). Therefore, we next tested whether excessive synaptic upscaling of GluA1 via DR was associated with the severity of neuroinflammation in *Ppt1^−/−^* mice.

**Figure 5:**
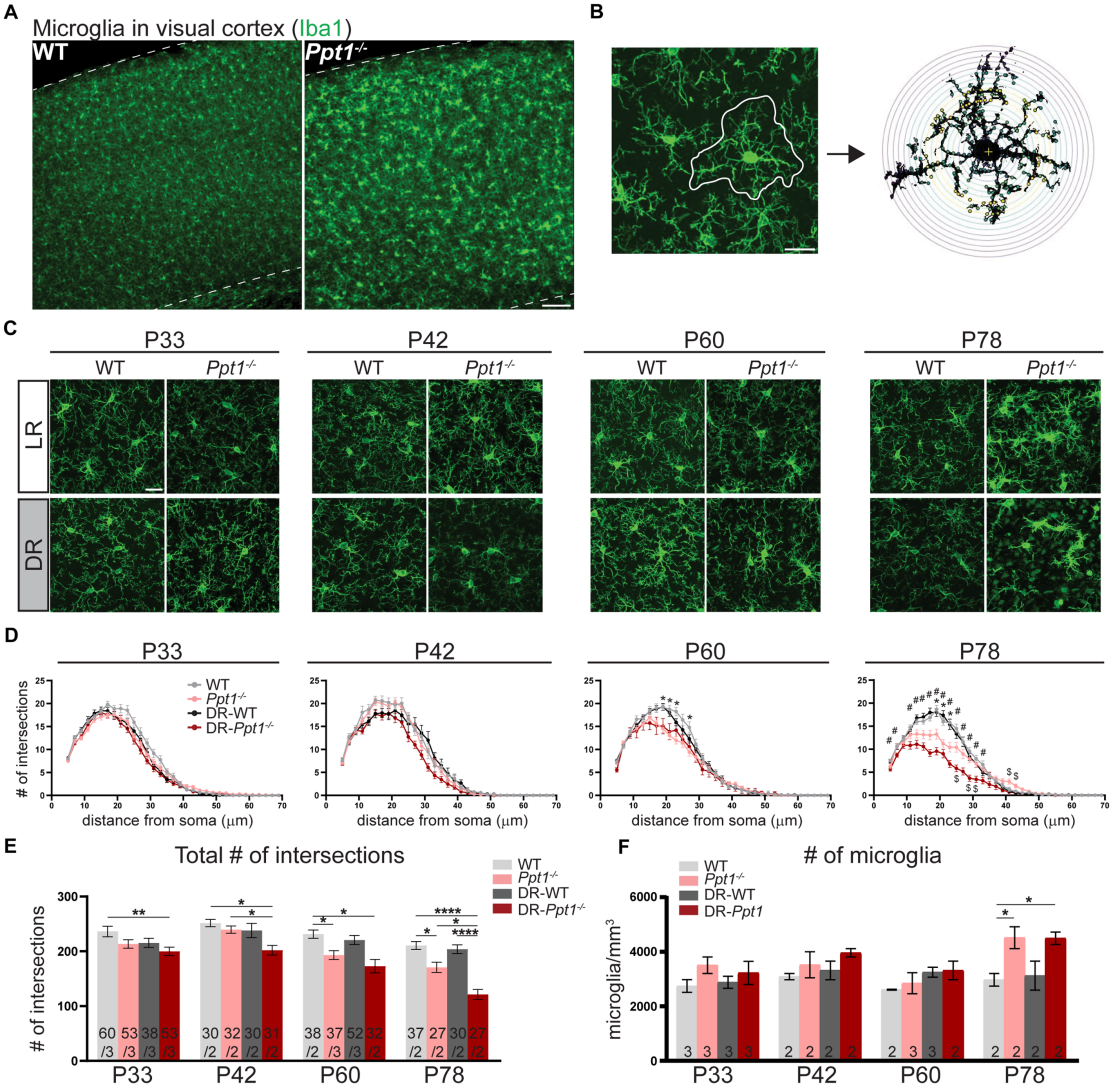
Induction of synaptic upscaling *in vivo* exacerbates neuroinflammation in *Ppt1^−/−^* visual cortex. **(A)** Representative low magnification images of Iba1 immunostaining in the visual cortex of WT and *Ppt1^−/−^* visual cortex at P78. Scale bar = 100 μm. **(B)** Representative thresholded microglia image and Sholl analysis overlay. Scale bar = 20 μm. **(C)** Representative high magnification images of Iba1 immunostaining for visual cortical (layer 2/3) microglia in WT, *Ppt1^−/−^*, DR-WT, and DR-*Ppt1^−/−^* mice across age (P28–P78). Scale bar = 20 μm. **(D)** Quantification of microglia branching by Sholl analysis in WT, *Ppt1^−/−^*, DR-WT, and DR-*Ppt1^−/−^* mice across age, where the number of intersections (*y*-axis) are averaged across cells at the specified distance from the soma (*x*-axis). Multiple *t*-tests: *p < 0.005 WT vs. *Ppt1^−/−^*; #*p* < 0.005 WT vs. DR-*Ppt1^−/−^*; $*p* < 0.005 *Ppt1^−/−^* vs. DR-*Ppt1^−/−^* (see Table 1). *N* = 27–60 cells, 2–3 animals per group. Number of cells/animals for each group is listed in panel **(E)**. **(E)** Quantification of the total number of intersections for each group of animals across age. Number of cells/animals in each group is listed on the graph. Two-way ANOVA: interaction of condition (genotype and rearing) × age (*F*(9, 522) = 2.098, *p = 0.0282); main effect of age (*F*(3, 522) = 21.42, ****p < 0.0001); main effect of genotype/rearing (*F*(3, 522) = 22.39, ****p < 0.0001). Tukey’s multiple comparisons indicated on the graph: **p = 0.0017 WT P33 vs. DR-*Ppt1^−/−^* P33; P42 *p = 0.0160 WT P42 vs. DR-*Ppt1^−/−^* P42; *p = 0.0250 *Ppt1^−/−^* P42 vs. DR-*Ppt1^−/−^* P42; *p = 0.0458 WT P60 vs. *Ppt1^−/−^* P60; *p = 0.0107 WT P60 vs. DR-*Ppt1^−/−^* P60; *p = 0.0197 DR-WT P60 vs. DR-*Ppt1^−/−^* P60; *p = 0.0178 WT P78 vs. *Ppt1^−/−^* P78; ****p < 0.0001 WT P78 vs. DR-*Ppt1^−/−^* P78; **p = 0.0040 *Ppt1^−/−^* P78 vs. DR-*Ppt1^−/−^* P78; ****p < 0.0001 DR-WT P78 vs. DR-*Ppt1^−/−^* P78. **(F)** Quantification of the number of microglia in a 1 mm^3^ volume of cortical tissue for each group of animals across age. The number of animals in each group is listed on the graph. Two-way ANOVA: no significant interaction (*F*(9, 22) = 1.288, *p* = 0.2976); main effect of age (*F*(3, 22) = 4.618, *p = 0.0119); main effect of genotype/rearing (*F*(3, 22) = 6.134, **p = 0.0034). Tukey’s multiple comparisons indicated on the graph: *p = 0.0247 WT P78 vs. *Ppt1^−/−^* P78; *p = 0.0268 WT P78 vs. DR-*Ppt1^−/−^* P78.

We immunostained LR-WT, LR-*Ppt1^−/−^*, DR-WT, and DR-*Ppt1^−/−^* brains for the microglia marker, Iba1 (Figure 5A), and performed morphological (i.e., Sholl) analysis on individual cells in the visual cortex (Figures 5B,C), since a classical sign of inflammatory microglial activation is a loss of ramification and ameboid shape (Giulian, 1987; Gehrmann et al., 1995). At P33 and P42, there were no differences between LR-WT and LR-*Ppt1^−/−^* cells in either the number of intersections as a function of distance from the cell soma (Figure 5D) or in terms of the total number of intersections (Figure 5E). By P60, however, there was a significant reduction in the number of intersections in LR-*Ppt1^−/−^* mice compared to LR-WT that persisted to P78, suggesting microglial morphology analysis is a sensitive measure of the neuroinflammatory phenotype in CLN1 mice (Figures 5D,E).

Importantly, as early as P33 in the visual cortex, DR-*Ppt1^−/−^* microglia exhibited a decrease in the total number of intersections compared to WT cells (Figure 5E). At P42, before any neurological deficit was detected in LR-*Ppt1^−/−^* mice, DR-*Ppt1^−/−^* mice exhibited significantly reduced microglial processes compared to LR-WT and DR-WT. Moreover, LR-*Ppt1^−/−^* animals (Figure 5D,E) demonstrated an acceleration of the neuroinflammatory phenotype in DR animals. This effect was even more robust at P78 (Figure 5D,E). Similar to previous findings (Sadhukhan et al., 2021), we also detected an increase in the number of microglia in LR-*Ppt1^−/−^* mice compared to WT counterparts at P78, though this effect was not exacerbated by DR (Figure 5F). These data demonstrate that DR worsens neuroinflammation in the visual cortex of *Ppt1^−/−^* mice.

Gliosis and neurodegeneration follow a systematic pattern in CLN1, affecting the thalamus (especially the visual thalamus) before the cortex (Kielar et al., 2007). Therefore, we also examined the neuroinflammatory phenotype of microglia in the dorsal lateral geniculate nucleus (dLGN) at ~P33 before detectable changes occurred in the cortex (Supplementary Figures 3A–C). Microglia in this region are crucial for synaptic refinement during the development of the visual circuit (Shatz, 1990; Katz and Shatz, 1996), so we focused our analysis on microglia at the borders of ipsilateral and contralateral retinal projections reaching the dLGN. We found a subtle but significant change in microglia morphology that suggests an emerging neuroinflammatory phenotype in the dLGN of LR-*Ppt1^−/−^* mice (Supplementary Figures 3A–C).

### 3.6 *In vivo* induction of synaptic upscaling exacerbates CLN1 disease symptoms

The regulation of synaptic calcium homeostasis, including calcium entry through CP-AMPARs, is crucial for neuronal health and prevention of epileptogenesis (McNamara et al., 2006). In addition, gliosis is not only a histopathological correlate of CLN1 disease progression but is associated with the onset of global symptoms, like seizures, in CLN1 mouse models (Zhang et al., 2022), where it may play a causative role (Vezzani et al., 2022). Having demonstrated an exaggerated synaptic upscaling phenotype, which alters neuronal calcium fluctuation (Koster et al., 2023), as well as exacerbated gliosis in DR-*Ppt1^−/−^* animals, we next examined whether these pathophysiological changes influenced CLN1 disease severity.

**Figure 6:**
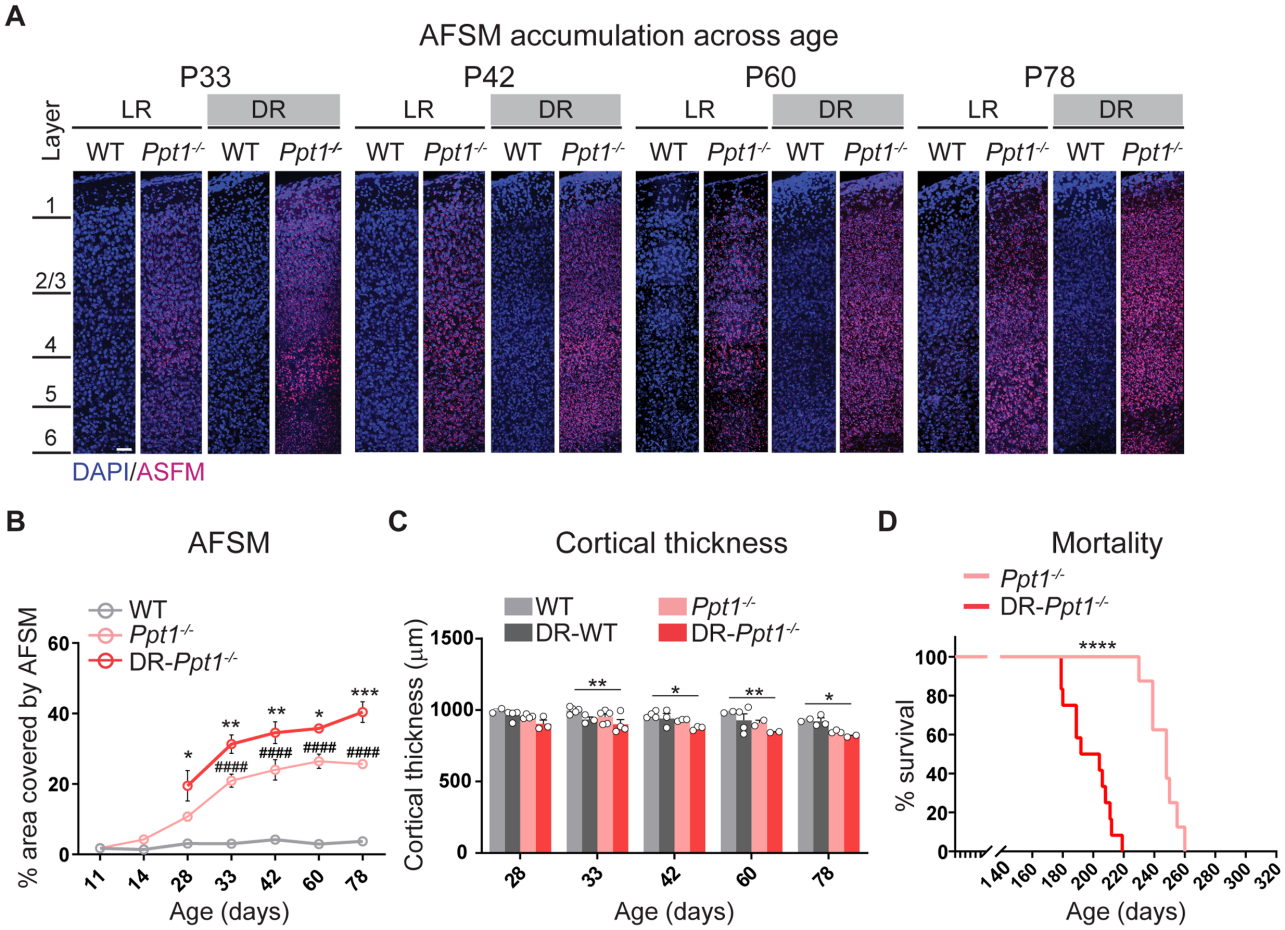
Induction of synaptic upscaling *in vivo* exacerbates disease pathology in *Ppt1^−/−^* mice. **(A)** Representative mid-sagittal sections of the medial visual cortex from LR-WT, LR-*Ppt1^−/−^*, DR-WT, and DR-*Ppt1^−/−^* mice showing the accumulation of AFSM with age (postnatal ages 33–78). Scale bar = 50 μm. **(B)** Quantification of the percent area covered by AFSM. Note that WT and LR-*Ppt1^−/−^* data are derived from Koster et al. (2019), but the histology and comparisons were all performed in parallel. Two-way ANOVA: interaction condition (genotype and rearing) × age (*F*(8, 41) = 3.072, **p = 0.0084); main effect of genotype/rearing (*F*(2, 41) = 174.5, ****p < 0.0001); main effect of age (*F*(4, 41) = 13.23, ****p < 0.0001). Tukey’s multiple comparison indicated on the graph: *p = 0.0157 P28 LR-*Ppt1^−/−^* vs. P28 DR-*Ppt1^−/−^*; **p = 0.0034 P33 LR-*Ppt1^−/−^* vs. P33 DR-*Ppt1^−/−^*; **p = 0.0030 P42 LR-*Ppt1^−/−^* vs. P42 DR-*Ppt1^−/−^*; *p = 0.0375 P60 LR-*Ppt1^−/−^* vs. P60 DR-*Ppt1^−/−^*. ***p = 0.0007 P78 LR-*Ppt1^−/−^* vs. P78 DR-*Ppt1^−/−^*. Data represent mean ± SEM. *N* = 3–4 animals/group. **(C)** Quantification of cortical thickness across age in WT, DR-WT, *Ppt1^−/−^*, and DR-*Ppt1^−/−^* mice. Two-way ANOVA: no interaction of condition (genotype and rearing) × age (*F*(12, 43) = 0.4683, *p* = 0.9223); main effect of genotype/rearing (*F*(3, 43) = 14.96, ****p < 0.0001); main effect of age (*F*(4, 43) = 5.330, **p = 0.0014). Tukey’s multiple comparison (simple effect within age) indicated on the graph: **p = 0.0046 P33 LR-WT vs. P33 DR-*Ppt1^−/−^*; *p = 0.0241 P42 LR-WT vs. P42 DR-*Ppt1^−/−^*; **p = 0.0078 P60 LR-WT vs. P60 DR-*Ppt1^−/−^*; *p = 0.0466 P78 LR-WT vs. P78 DR-*Ppt1^−/−^*; *p = 0.0401 P78 DR-WT vs. P78 DR-*Ppt1^−/−^*. Number of animals for each group (*N* = 2–5) is displayed on the graph (individual points). Data represent mean ± SEM. **(D)** Kaplan–Meier plot of mortality in LR-*Ppt1^−/−^* and DR-*Ppt1^−/−^* mice. Log-rank (Mantel-Cox) test: ****p < 0.0001 LR-*Ppt1^−/−^* vs. DR-*Ppt1^−/−^*. N = 6 *Ppt1^−/−^*, 10 for DR-*Ppt1^−/−^*.

Indeed, concomitant with excessive synaptic upscaling, the accumulation of AFSM increased in the visual cortex (Figures 6A,B). Furthermore, although dark rearing is known to slightly reduce visual cortical thickness in WT animals (Fifková, 1970; Takács et al., 1992), we observed a statistically significant reduction only in DR-*Ppt1^−/−^* mice compared to WT animals raised under standard conditions (Figure 6C). Importantly, mortality occurred significantly earlier because of severe seizures (see Materials and Methods) in DR-*Ppt1^−/−^* animals (Figure 6D). Thus, the lack of Ppt1-mediated depalmitoylation drives an exaggerated synaptic upscaling of GluA1 and enhanced neuroinflammation *in vivo* that corresponds to exacerbated CLN1 pathology. These results are consistent with a substantive connection between aberrant synaptic plasticity and CLN1 disease progression; however, further experiments are required to determine the causative agents (i.e., does excessive CP-AMPAR upregulation itself worsen disease).

### 3.7 FK506 treatment subtly improves neuroinflammation in *Ppt1^−/−^* mice

**Figure 7:**
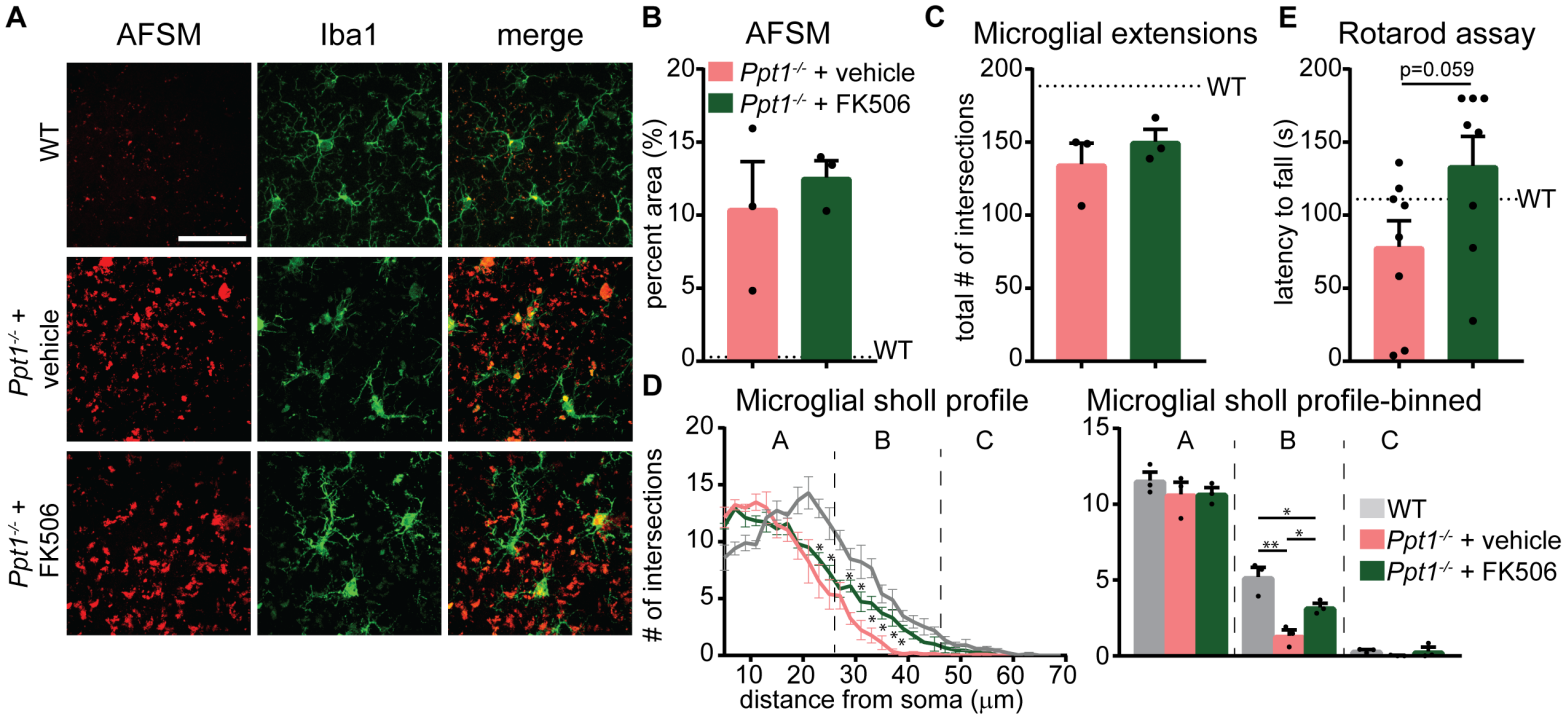
FK506 treatment subtly improves neuroinflammation in *Ppt1^−/−^* mice. **(A)** Representative images of AFSM accumulation (red) and Iba-immunostaining of microglia (green) in the visual cortex of WT, vehicle-treated *Ppt1^−/−^*, and FK506-treated *Ppt1^−/−^* mice at 4 months of age. Scale = 50 μm. **(B)** Quantification of the percent area occupied by AFSM puncta in WT (represented by dashed horizontal line), vehicle-treated *Ppt1^−/−^*, and FK506-treated *Ppt1^−/−^* mice. **(C)** Quantification of the total number of microglial intersections with a Sholl analysis, a measure of microglial ramification, in WT (represented by dashed horizontal line), vehicle-treated *Ppt1^−/−^*, and FK506-treated *Ppt1^−/−^* mice. **(D)** *Left*, Sholl profile of microglial ramification in WT, vehicle-treated *Ppt1^−/−^*, and FK506-treated *Ppt1^−/−^* mice. *p < 0.05 vehicle-treated *Ppt1^−/−^* mice vs. FK506-treated *Ppt1^−/−^* mice at each distance from the soma by *t*-tests. See Table 3 for full statistics. *Right*, the same data shown in the Sholl profile but represented as three bins [near, intermediate, and distant from the soma, represented by panels **(A–C)**, respectively]. One-way ANOVA within bin B: *F*(2, 6) = 18.19, **p = 0.0028. Holm–Sidak multiple comparisons on the graph: *p = 0.0028 WT vs. vehicle-treated *Ppt1^−/−^* mice, *p = 0.0413 WT vs. FK506-treated *Ppt1^−/−^* mice, *p = 0.0413 vehicle-treated *Ppt1^−/−^* mice vs. FK506-treated *Ppt1^−/−^* mice. **(E)** Quantification of rotarod performance in WT (represented by dashed horizontal line), vehicle-treated *Ppt1^−/−^*, and FK506-treated *Ppt1^−/−^* mice at 4 months. *p* = 0.059 vehicle-treated *Ppt1^−/−^* mice vs. FK506-treated *Ppt1^−/−^* mice by *t*-test.

Considering that the Akap5 complex is dysregulated in the *Ppt1^−/−^* brain, NFAT activation is sensitized in upscaled *Ppt1^−/−^* neurons, and *in vivo* induction of synaptic upscaling exacerbates disease symptoms, we reasoned that suppressing calcineurin activity should alleviate CLN1 progression. Therefore, we treated *Ppt1^−/−^* animals from 1 to 4 months of age with the calcineurin inhibitor, FK506 (3 mg/kg dissolved in drinking water), which is an FDA-approved immunosuppressant under the trade names Tacrolimus or Prograf. Passive administration of FK506 in *Ppt1^−/−^* mice demonstrated no effect on the degree of AFSM accumulation (Figures 7A,B). In addition, FK506 did not significantly suppress microglial activation as measured by the total number of intersections with a Sholl analysis (Figure 7C). However, further scrutiny of the data indicated that while inflammatory activation of microglia in both vehicle- and FK506-treated *Ppt1^−/−^* mice was robust, FK506 treatment suppressed the transformation of microglia into a fully ameboid phenotype (Figure 7A). Accordingly, comparison of the Sholl profile (Figure 7D, left) revealed a greater number of microglia with ramifications at intermediate distances from the cell soma, which were largely absent in vehicle-treated mice (Figure 7D, right). Finally, as motor dysfunction is a primary symptom of CLN1 in humans and mice (Gupta et al., 2003; Dearborn et al., 2015; Nita et al., 2016), we next tested whether FK506 treatment improves motor symptoms in *Ppt1^−/−^* mice using a Rotarod assay. Under these conditions, FK506 treatment appeared to slightly improve motor behavior compared to vehicle-treated *Ppt1^−/−^* counterparts, though the effect did not quite reach statistical significance (*p* = 0.059, Student’s *t*-test vs. vehicle-treated *Ppt1^−/−^* mice) (Figure 7E). Collectively, these results show that suppressing calcineurin activity via FK506 treatment may modestly slow neuroinflammatory progression in *Ppt1^−/−^* mice.

## 4 Discussion

We demonstrate herein that synaptic upscaling of GluA1 is exaggerated in DR-*Ppt1^−/−^* mice and correlates with an acceleration of disease progression that includes increased neuroinflammation and earlier mortality. Palmitoyl-proteomics profiling revealed a widespread, albeit subtle, over-palmitoylation of many synaptic proteins in *Ppt1^−/−^* visual cortical synaptosomes and implicated the scaffolding protein Akap5 and its associated signaling molecules. Further scrutiny of this pathway demonstrated that NFAT activation is increased in *Ppt1^−/−^* neurons in response to neuronal stimulation, particularly through CP-AMPARs, and provoked us to test the efficacy of FK506 oral administration in *Ppt1^−/−^* mice. Passive administration of FK506 provided subtle histopathological benefits, emphasizing the need for larger cohort studies employing FK506 in CLN1 mouse models. Taken together, our data reveal Akap5 as a potential mechanistic link between initial disease-driven changes to synaptic function and downstream neuroinflammation in CLN.

There are several limitations to the current study. For instance, we did not examine whether GluA1 palmitoylation increased with induction of synaptic upscaling in DR animals, and therefore did not corroborate our previous *in vitro* findings (Koster et al., 2023). Similarly, it will be important to know whether Akap5 palmitoylation increases during synaptic scaling. Surely, future experiments on the relationship between synaptic protein palmitoylation and synaptic scaling should prioritize these molecules using manipulations that allow a causative interpretation (e.g., palmitoylation-deficient Akap5), which we failed to do. Furthermore, by performing palmitoyl-proteomics at a single timepoint, particularly in young mice, we failed to capture statistically significant changes to the palmitoylation of synaptic proteins in *Ppt1^−/−^* animals. Fortunately, the analyses pointed to Akap5 and associated pathways as being dysregulated in *Ppt1^−/−^* brains, but we cannot rule out that these analyses may have missed additional, perhaps equally important, signaling pathways that underpin the progression of CLN1. Indeed, protein kinase A is also anchored by Akap5 and known to regulate synaptic scaling (Goel et al., 2011; Diering et al., 2014), representing an attractive target for future studies on how Ppt1 activity might influence AMPAR trafficking. Finally, although we demonstrate that excessive synaptic upscaling in *Ppt1^−/−^* neurons is associated with an exacerbation of disease features *in vivo*, further experiments are required to directly tie these phenomena together. Below, we focus our discussion on the positive data we were able to obtain.

### 4.1 Palmitoyl-proteomics links synaptic dysfunction to neuroinflammation

While we maintain that over-palmitoylation of GluA1 likely contributes to the increased incorporation of CP-AMPARs at upscaled *Ppt1^−/−^* synapses (Koster et al., 2023), we demonstrate here that Akap5 is also hyperpalmitoylated in *Ppt1^−/−^* synaptosomes. Thus, the increased synaptic incorporation of GluA1 during upscaling may also arise from enhanced scaffolding by Akap5. Previous experiments implicate Akap5 in the regulation of CP-AMPARs during synaptic scaling (Sanderson et al., 2018). In addition, palmitoylation of Akap5 is required for the postsynaptic insertion of CP-AMPARs during LTP (Purkey et al., 2018); although this study also notes that palmitoylated Akap5 limits the synaptic incorporation of CP-AMPARs under basal conditions, suggesting that Akap5 palmitoylation differentially effects CP-AMPAR trafficking dynamics at a steady state compared to bouts of plasticity. Akap5 also requires depalmitoylation for its removal from the postsynaptic site and undergoes ubiquitination to downregulate GluA1-containing AMPARs during chemical LTD (Woolfrey et al., 2018; Cheng et al., 2020). Therefore, we anticipate that Akap5 normally undergoes depalmitoylation-dependent degradation (Cheng et al., 2020) and that this mechanism is diminished in *Ppt1^−/−^* neurons. Consequently, we postulate that overly palmitoylated Akap5 harbors an enlarged perisynaptic pool of CP-AMPARs that is mobilized during synaptic upscaling in *Ppt1^−/−^* neurons either at the extrasynapse or dendritic endosomes (He et al., 2009; Diering et al., 2014). Recent detailed analysis of the synaptic and perisynaptic localization of palmitoylated Akap5 agrees with such a notion (Chen et al., 2022).

Our data also indicate that the over-palmitoylation of Akap5 leads to a sensitization of the NFAT transcriptional program in *Ppt1^−/−^* neurons. Not only does this provide a mechanistic link between synaptic alterations and gliosis in CLN1 (Jalanko et al., 2005; Kielar et al., 2007; Macauley et al., 2009, 2011, 2014), but, in addition, the calcineurin-NFAT pathway itself mediates synaptic scaling through the turnover of CP-AMPARs (Kim and Ziff, 2014). Therefore, mis-localization of Akap5 or impaired interactions with calcineurin due to loss of Ppt1 likely drives over-activation of NFAT-dependent transcription, causing a vicious cycle of exaggerated upscaling of CP-AMPARs in the *Ppt1^−/−^* brain. In sum, several related mechanisms may collaborate to drive exaggeration of CP-AMPAR incorporation during synaptic scaling up in *Ppt1^−/−^* neurons.

### 4.2 Dark rearing exacerbates CLN1 pathology: contribution of exaggerated synaptic upscaling

One motivation for employing the DR paradigm in *Ppt1^−/−^* mice was brought on by previous work, which demonstrated that dark-rearing mouse models of other developmental disorders, like Rett and Angelman syndromes, mitigated disease symptoms (Yashiro et al., 2009; Durand et al., 2012). Accordingly, we originally anticipated an improvement in CLN1 pathophysiology in DR-*Ppt1^−/−^* mice. However, our study demonstrates that DR-*Ppt1^−/−^* animals have the opposite effect, exacerbating several markers of disease progression, including mortality. Why might this be the case?

Before the demonstration of its beneficial effects in neurodevelopmental disease models, DR was established as a model of synaptic upscaling *in vivo* (Goel et al., 2006, 2011; Goel and Lee, 2007). Here, we corroborate these data by demonstrating that DR of *Ppt1^−/−^* mice exaggerates synaptic upscaling of GluA1, extending our previous findings (Koster et al., 2023) that used exogenous manipulations (e.g., TTX induction of upscaling). This is precisely the opposite effect conferred by loss of either Ube3a (Angelman syndrome) or MeCP2 (Rett syndrome), which suppresses upscaling (Blackman et al., 2012; Qiu et al., 2012; Pastuzyn and Shepherd, 2017). Therefore, we postulate that the opposing effects of DR on disease pathology in CLN1 versus Rett or Angelman syndrome mice arise from the differential impact that the proteins mutated in these conditions have on synaptic scaling. In other words, whereas DR alleviated symptoms in Rett and Angelman syndrome mice in part by overcoming the stagnation of synaptic upscaling, this same manipulation proved detrimental in the CLN1 model because it facilitated an exaggerated synaptic upscaling. Still, taking the broader context reveals that several proteins implicated in neurodevelopmental disorders regulate the common pathway of synaptic scaling and imply that normalizing synaptic scaling dynamics might alleviate disease symptoms.

### 4.3 Implications for CLN1 progression and therapeutic intervention

The sensitization of calcineurin-NFAT activity in *Ppt1^−/−^* neurons represents a new therapeutic target in CLN1 that has a history of being inhibited in other conditions requiring immunosuppression with FDA-approved drugs such as Tacrolimus (FK506) and cyclosporin. Drug repurposing is a major priority of the FDA (along with the incentives for orphan drug discovery) due to the economics and relative speed of getting potential therapeutics to patients relative to the traditional approach, which typically takes hundreds of millions of dollars and 10 or more years. Therefore, our demonstration here that FK506 has beneficial effects in CLN1 mice is a substantial first step toward its use in the clinic for patients suffering from CLN1. Although we observed quite a modest effect here, we offer the following caveat. Our aim was to perform a small pilot study of FK506 efficacy that employed several readouts, not just histology or behavior, but both, and as such, we chose a terminal time point for our cohort of 4 months with the hope of capturing such an effect. However, motor deficits in *Ppt1^−/−^* mice emerge between 3 and 5 months, often reaching statistical significance by the 5-month mark (Macauley et al., 2009; Groh et al., 2021), while our own data show that neuroinflammation is robust by 3 months. Therefore, by attempting to capture the potential effects of FK506 across these two measures, we may have limited our power to detect either. Accordingly, our data warrant further, more detailed study of FK506 in CLN1.

Interestingly, Tacrolimus has demonstrated beneficial effects in multiple animal models of aging or neurodegeneration (Radhakrishnan et al., 2021; Fracassi et al., 2022; Sordo et al., 2022) and was the subject of an open-label clinical trial (NCT04263519). Moreover, human patients prescribed calcineurin inhibitors, including Tacrolimus, show reduced prevalence of developing Alzheimer’s disease (Silva et al., 2023). These studies suggest Tacrolimus or next-generation analogs may be a promising intervention in multiple neurodegenerative diseases. Future studies focused on testing the efficacy of FK506 or other calcineurin-directed therapeutics in a larger preclinical setting or in CLN1 patients will be of great interest.

In conclusion, we demonstrate here with an interdisciplinary approach how failed protein depalmitoylation disrupts synaptic plasticity via impaired proteostasis and triggers neuroinflammatory signaling that underlies a devastating disease. Importantly, these findings offer multiple novel targets for therapeutic intervention, at least one of which (calcineurin inhibitors) can benefit from the FDA and clinical priority to repurpose well-tolerated drugs and can therefore be more rapidly tested in suffering patients.

## Data availability statement

The original contributions presented in the study are publicly available. This data can be found at: https://massive.ucsd.edu/ProteoSAFe/dataset.jsp?task=e9c851fbf5214e1d8f257a9f390614e3.

## Ethics statement

The animal study was approved by University of Illinois of Chicago Institutional Animal Care and Use Committee. The study was conducted in accordance with the local legislation and institutional requirements.

## Author contributions

KK: Conceptualization, Data curation, Formal analysis, Investigation, Methodology, Visualization, Writing – original draft, Writing – review & editing. ZF: Formal analysis, Investigation, Writing – review & editing, Writing – original draft. TN: Formal analysis, Investigation, Methodology, Writing – review & editing, Writing – original draft. AN: Formal analysis, Writing – review & editing, Writing – original draft. LN-G: Formal analysis, Writing – review & editing, Writing – original draft. KW: Methodology, Resources, Writing – original draft, Writing – review & editing. MD: Methodology, Resources, Writing – original draft, Writing – review & editing. SC: Conceptualization, Methodology, Supervision, Writing – original draft, Writing – review & editing. AY: Conceptualization, Data curation, Formal analysis, Funding acquisition, Resources, Supervision, Writing – original draft, Writing – review & editing.

## Footnotes

^1^ https://string-db.org/

## In memoriam

This article is dedicated to Dr. Akira Yoshii, MD, PhD, who sadly passed away in March of 2023 during its preparation.

## Supplementary material

SUPPLEMENTARY FIGURE S1 SynGo and network analysis of palmitoyl-proteomics from visual cortical lysates. **(A)** Top 10 enriched SynGO terms from proteins increased 1.2-fold in *Ppt1^−/−^* visual cortical lysates. **(B)** Network analysis of the genes increased in *Ppt1^−/−^* lysates by 1.2-fold that were annotated with the top biological process SynGO term “process at the synapse.

SUPPLEMENTARY FIGURE S2 GluN2B palmitoylation is increased in *Ppt1^−/−^* visual cortical synaptosomes. Representative immunoblots (left) and quantification of the palmitoylated/non-palmitoylated ratio (normalized to β-actin, right) of GluN2B in APEGS-processed visual cortical synaptosomes at P42. *t*-test: **p* = 0.0396. *n* = 3 mice/group.

SUPPLEMENTARY FIGURE S3 Emerging neuroinflammation in dLGN of young *Ppt1^−/−^* mice. **(A)** Schematic of the injection paradigm for measurement of microglial morphology at the border between ipsilateral and contralateral retinogeniculate projections. **(B)** Representative images of CTB 488, CTB 555, and Iba1 immunostaining in the dLGN of a WT and *Ppt1^−/−^* mouse. Scale = 50 μm. **(C)** Sholl analysis profile of microglia (Iba1) morphology in WT and *Ppt1^−/−^* dLGN between P21 and P42 (see Table 2).
