# Supplementary figures and images for: Akap5 links synaptic dysfunction to neuroinflammatory signaling in a mouse model of infantile neuronal ceroid lipofuscinosis
Source: Front Synaptic Neurosci. 2024 May 10;16:1384625. doi: 10.3389/fnsyn.2024.1384625 (PMC11116793; doi:10.3389/fnsyn.2024.1384625)

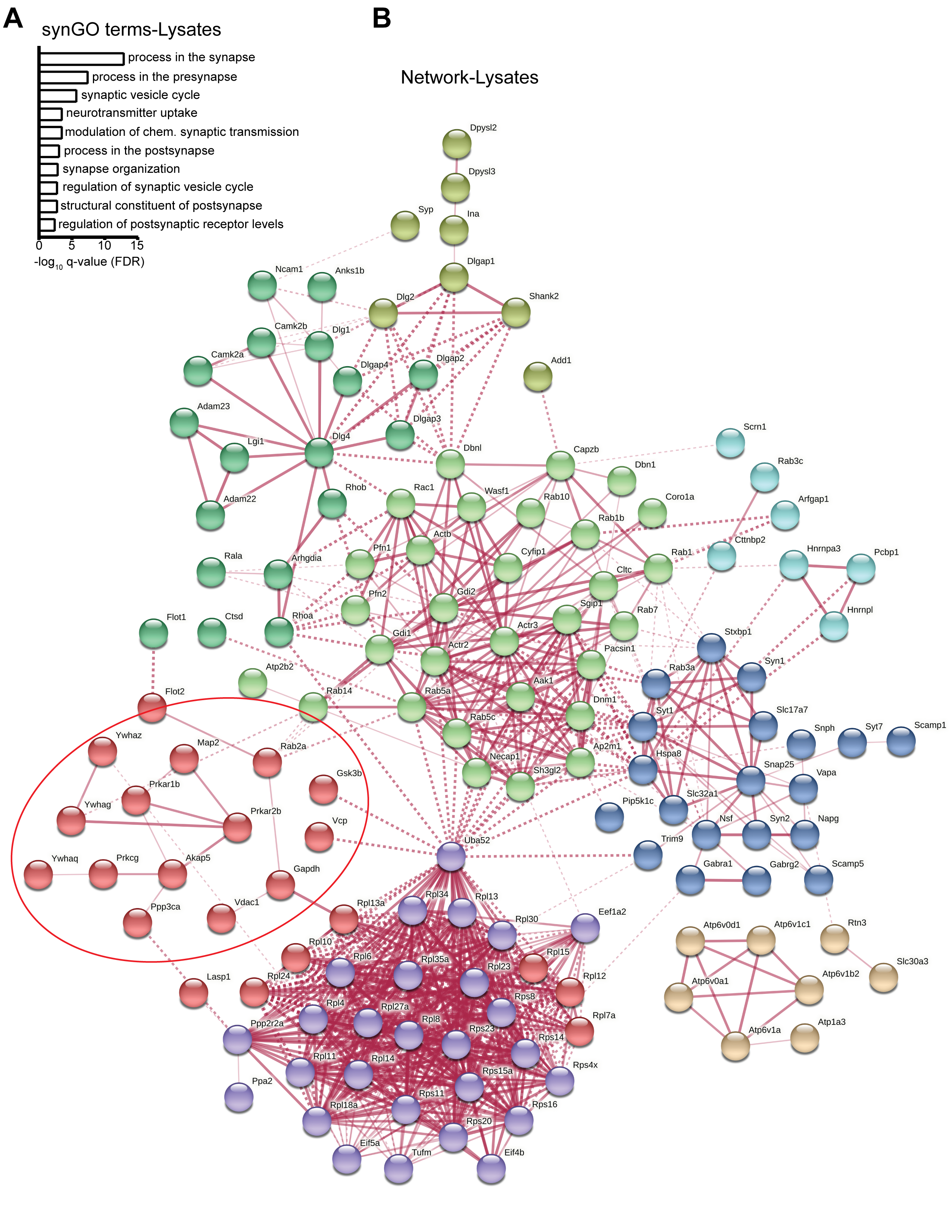

Supplement: SUPPLEMENTARY FIGURE S1 — SynGo and network analysis of palmitoyl-proteomics from visual cortical lysates. (A) Top 10 enriched SynGO terms from proteins increased 1.2-fold in Ppt1−/− visual cortical lysates. (B) Network analysis of the genes increased in Ppt1−/− lysates by 1.2-fold that were annotated with the top biological process SynGO term “process at the synapse. [file Image_1.TIF]

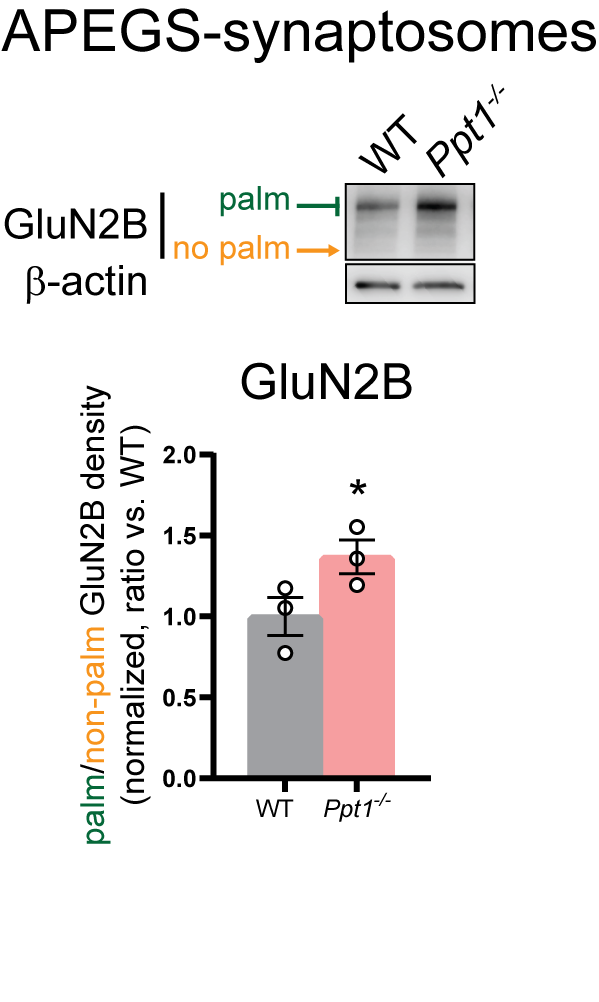

Supplement: SUPPLEMENTARY FIGURE S2 — GluN2B palmitoylation is increased in Ppt1−/− visual cortical synaptosomes. Representative immunoblots (left) and quantification of the palmitoylated/non-palmitoylated ratio (normalized to β-actin, right) of GluN2B in APEGS-processed visual cortical synaptosomes at P42. t-test: *p = 0.0396. n = 3 mice/group. [file Image_2.tif]

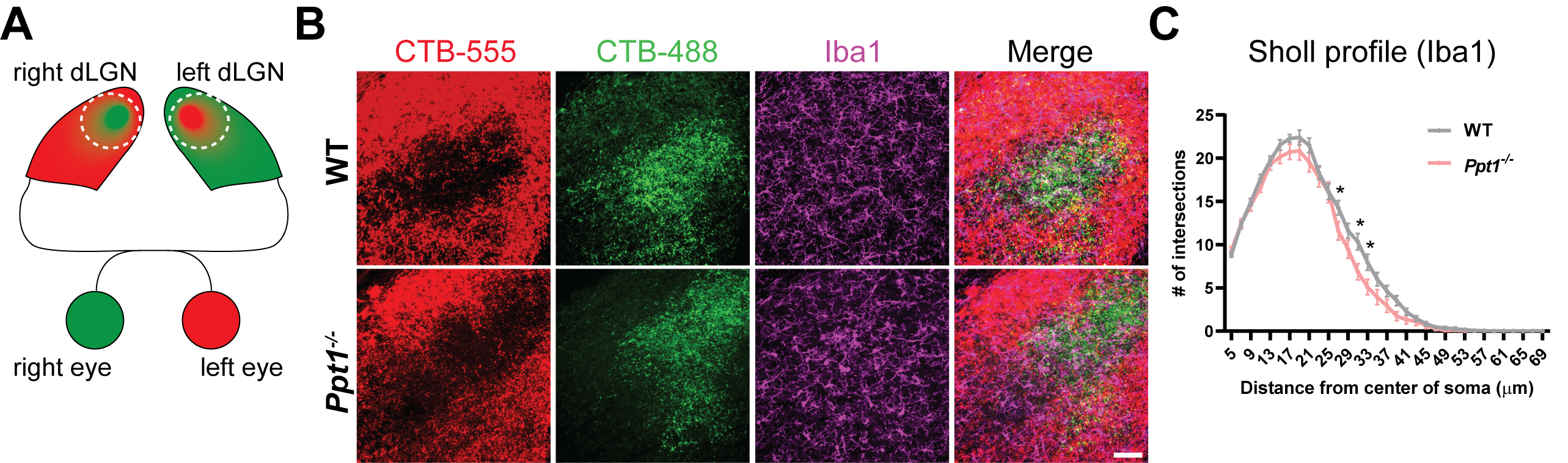

Supplement: SUPPLEMENTARY FIGURE S3 — Emerging neuroinflammation in dLGN of young Ppt1−/− mice. (A) Schematic of the injection paradigm for measurement of microglial morphology at the border between ipsilateral and contralateral retinogeniculate projections. (B) Representative images of CTB 488, CTB 555, and Iba1 immunostaining in the dLGN of a WT and Ppt1−/− mouse. Scale = 50 μm. (C) Sholl analysis profile of microglia (Iba1) morphology in WT and Ppt1−/− dLGN between P21 and P42 (see Table 2). [file Image_3.TIF]
